# Transcriptome changes during the initiation and progression of Duchenne muscular dystrophy in *Caenorhabditis elegans*

**DOI:** 10.1093/hmg/ddaa055

**Published:** 2020-03-30

**Authors:** Heather C Hrach, Shannon O’Brien, Hannah S Steber, Jason Newbern, Alan Rawls, Marco Mangone

**Affiliations:** 1 Molecular and Cellular Biology Graduate Program, School of Life Sciences, 427 East Tyler Mall, Tempe, AZ 85287 4501, USA; 2 Virginia G. Piper Center for Personalized Diagnostics, The Biodesign Institute at Arizona State University, 1001 S McAllister Ave, Tempe, AZ 85281, USA; 3 Barrett Honors College, Arizona State University, 751 E Lemon Mall, Tempe, AZ 85281, USA; 4 School of Life Sciences, 427 East Tyler Mall, Tempe, AZ 85287 4501, USA

## Abstract

Duchenne muscular dystrophy (DMD) is a lethal, X-linked disease characterized by progressive muscle degeneration. The condition is driven by nonsense and missense mutations in the dystrophin gene, leading to instability of the sarcolemma and skeletal muscle necrosis and atrophy. Resulting changes in muscle-specific gene expression that take place in dystrophin’s absence remain largely uncharacterized, as they are potentially obscured by the chronic inflammation elicited by muscle damage in humans. *Caenorhabditis elegans* possess a mild inflammatory response that is not active in the muscle, and lack a satellite cell equivalent. This allows for the characterization of the transcriptome rearrangements affecting disease progression independently of inflammation and regeneration. In effort to better understand these dynamics, we have isolated and sequenced body muscle-specific transcriptomes from *C. elegans* lacking functional dystrophin at distinct stages of disease progression. We have identified an upregulation of genes involved in mitochondrial function early in disease progression, and an upregulation of genes related to muscle repair in later stages. Our results suggest that in *C. elegans*, dystrophin may have a signaling role early in development, and its absence may activate compensatory mechanisms that counteract muscle degradation caused by loss of dystrophin. We have also developed a temperature-based screening method for synthetic paralysis that can be used to rapidly identify genetic partners of dystrophin. Our results allow for the comprehensive identification of transcriptome changes that potentially serve as independent drivers of disease progression and may in turn allow for the identification of new therapeutic targets for the treatment of DMD.

## Teaser

One Sentence Summary A tissue-specific transcriptome analysis of dystrophin-deficient muscle in *C. elegans* reveals that dystrophin has distinct, dynamic signaling roles in early and late-stage progression of DMD.

## Introduction

Duchenne muscular dystrophy (DMD) is an X-linked, recessive disease caused by out of frame mutations in the dystrophin gene (1). The dystrophin gene codes for a structural protein found beneath the sarcolemma, where it is anchored both to the dystrophin glycoprotein complex (DGC) and cytoskeletal actin, thus stabilizing the protein complex and the integrity of the cell membrane (2). In humans, the absence of functional dystrophin results in progressive degeneration of the skeletal and cardiac muscles. The hallmark symptoms of DMD extend beyond muscle degeneration to include respiratory failure, cardiomyopathy, pseudohypertrophy and chronic, widespread inflammation of the muscle. The condition remains the most commonly diagnosed type of muscular dystrophy, affecting approximately 1 in 3500 live male births globally.

While the role of dystrophin in forming a physical connection between the extracellular matrix and cytoskeleton has been well characterized (3), a comprehensive molecular definition of dystrophin’s function is not fully understood. Outside of dystrophin’s structural role, decades of research have led to the proposal that dystrophin has an essential signaling role in the muscle as well, and its absence may induce a myriad of changes in gene expression that in turn influence the progression of DMD symptoms. Dystrophin deficiencies have been implicated in changes in neuronal nitric oxide synthase (nNOS) localization (4–6), miRNA expression (7, 8), and alterations to the Wnt, and Hippo signaling pathways (9, 10). Despite all the progress made in elucidating the signaling role of dystrophin, we still do not have an all-encompassing definition of the signaling consequences of dystrophin deficiencies. This issue is due in part to the fact that chronic inflammation of mammalian muscle has the ability to obscure more subtle changes in gene expression outside of the inflammatory pathway.

Vertebrate models of DMD include the *mdx* mouse (11) and the golden retriever muscular dystrophy canine (12). Both models have contributed significantly towards our understanding of dystrophin’s function (13), but face limitations when studying the cell autonomous effects of dystrophin deficiencies on skeletal muscle, independently of the myolysis and fibrosis associated with chronic inflammation observed in mammals. In addition, *mdx* mice are challenging to use for the study of molecular mechanisms using high-throughput approaches such as genome-wide screens and other large-scale studies (14).

Mitochondrial dysfunction and systemic deregulation of cellular energy homeostasis have been observed in DMD patients, *mdx* mice and the invertebrate *Caenorhabditis elegans* (15–19). Mitochondria play an important role in both healthy and dystrophic muscle function, as a steady-state flux of ATP, Ca+ and other components of energy metabolism are required for muscle contraction. A recent study identified a significant increase in enzymes that are major consumers of NAD+ in dystrophic muscle tissues*,* and its replenishment was shown to ameliorate symptoms in *mdx* mice and *C. elegans* (17). Mitochondria also play a pivotal role in disease progression, but it is still not clear if their dysfunction is induced by the damage of muscle fibers, which in turn induce muscle paralysis and necrosis, or if it occurs in early stages of the disease when paralysis and loss of muscle tissue have not yet been initiated (20). The field of DMD research has yet to reach a comprehensive definition of the exact role mitochondrial function plays in the initiation and progression of DMD.

The invertebrate model *C. elegans* has the potential to address these questions and serve as an informative model system for DMD. These nematodes possess a singular ortholog of the dystrophin gene (*dys-1*), which is similar in protein size to the human dystrophin gene and contains similar actin-binding and scaffolding regions (21). Additionally, several essential members of the DGC are conserved between humans and nematodes (22, 23). The *C. elegans* DGC is comparable with the human complex and includes key proteins such as dystrobrevin (DYB-1), sarcoglycans (SGCA-1, SGCB-1 and SGN-1) and the syntrophins (STN-1 and STN-2) (24). This predicts strong selective pressure for functional conservation of the DGC from *C. elegans* to humans.

The *C. elegans* mutant strains *dys-1(cx18) and dys-1(eg33)* represent a novel tool to study DMD *in vivo* (21, 25). These mutant strains contain different nonsense mutations causing a premature stop codon in different portions of the gene. DYS-1 has been well characterized for its function in the worm body wall and vulva muscles, and its expression has been detected in a number of tissues outside of the muscle, including the gonad and pharynx (26). Mimicking the human condition, early reports have shown that loss of *dys-1* does not result in dramatic muscular degeneration phenotypes. Instead, they display defects in motility that are phenotypically distinct from humans, but are still the direct result of a loss of functional *dys-1.* This includes anomalous bending of the head, hyperactivity, impaired burrowing ability, hyper contraction of the body wall muscle and age-dependent, progressive loss of locomotor function (27). In later stages, *dys-1* worm strains also exhibit partial muscle cell death with shorter lifespans than *wt* worms (25) and momentary lack of forward progression in dystrophic worms cultivated within agar pipettes (27).

Both *dys-1* worm strains display similar disease phenotypes, suggesting that both mutations, although in different portions of the *dys-1* gene, affect similar pathways. The introduction of human *dystrophin* cDNA in these mutant worms rescues these phenotypes (25), suggesting they are indeed a highly appropriate disease model. Of note, *dys-1*(*eg33*) and *dys-1*(*cx18*) strains have been recently found to respond differently to stress and to pharmacological treatment (19), with the *dys-1(eg33)* strain being more phenotypically severe.

*C. elegans* possess an innate immune response; the adaptive immunity is primitive and cell-mediated immunity is absent (28). The absence of chronic inflammation and muscle regeneration, which are normally implicated in the progression of myofiber necrosis and fibrosis in human DMD patients and mammalian models, may be one component that allows these worm strains to move somewhat similarly to *wt* worms (29). Changes in motility are subtle, localized to specific areas, and become most apparent in adult worms. Because the muscle tissue in these strains is not being actively damaged by chronic inflammation as in patients with DMD and *mdx* mice, there are no major changes in muscle structure between *wt* and dystrophic muscles in *C. elegans*. This lack of extensive muscle damage or paralysis allows for the study of cell autonomous contributions to the progression of DMD symptoms and resulting changes in gene expression in the absence of inflammation.

Our lab has previously adapted an established technique named PAT-Seq, which optimizes tissue-specific RNA isolation from intact organisms (30), allowing the identification of tissue-specific transcriptomes at single-base resolution (31–33). This method, named PAT-Seq, takes advantage of the binding affinity and specificity of the cytoplasmic PolyA Binding Protein (PABPC1) to polyA tails of mRNAs (34) by fusing the worm ortholog of PABPC1 (*pab-1)* to a 3XFLAG tag and a fluorescent marker (GFP::*pab-1*::3xFLAG), and placing this cassette under the control of a tissue-specific promoter of choice. UV crosslinking and immunoprecipitation using α-FLAG antibodies in worms expressing this cassette allows the isolation and sequencing of tissue specific polyA+ RNA. The result is a high-quality tissue-specific transcriptome that is depleted of contaminating transcripts from outside tissues. Using this technique, our lab recently profiled *C. elegans* intestine, pharynx and body muscle tissues (31). This study allowed us to define and analyze body muscle transcriptome at an unprecedented resolution (31).

In order to identify muscle-specific transcriptome changes occurring throughout DMD progression, we crossed the *dys-1(cx18)* and *dys-1(eg33)* mutant strains with our *myo-3p*::GFP::*pab-1*::3xFLAG, or PolyA-Pull (*wt* PAP) worm strain (31), producing two novel strains named DP1 and DP2, respectively. These two strains were then used to isolate and sequence muscle-specific transcriptomes in worms lacking the dystrophin protein at different stages of the disease, allowing us to fully define the dynamic transcriptome changes occurring in the absence of inflammation at controlled time points, during early and late stages of symptom progression. Furthermore, we have examined changes in gene expression that are unique to each of the dystrophin-deficient strains in order to better understand functional differences between the two mutant strains. We have also performed targeted genetic experiments to study the contribution of selected genes towards symptom progression identified by our approach.

We have found that that the two different truncated versions of the dystrophin gene in *dys-1*(*cx18*) and *dys-1*(*eg33*) strains are both stably transcribed and drive distinct differences in gene expression pattern. Our transcriptome analyses have found that the absence of *wt* dystrophin in *C. elegans* lead to widespread splicing errors, and initiate deregulation of mitochondrial function in the earliest stages of development. This impairment leads to differential expression of genes involved in muscle function and differentiation during later stages of development and adulthood, perhaps as a part of a compensatory mechanism that is able to impede dystrophin-dependent muscle degeneration.

## Results

### PAT-Seq on *dys-1 C. elegans* strains produced high-quality muscle-specific mRNAs

In order to sequence *C. elegans* dystrophin-deficient muscle tissue, we have taken advantage of the PAT-Seq assay used in our previous studies (31, 32). The original *wt* PAP strain expresses the gene *pab-1* fused to GFP (N-terminus) and a 3xFLAG tag (C-terminus) restricted to the muscle by the tissue-specific promoter *Pmyo-3* (31, 32). *pab-1* is the *C. elegans* ortholog of the human cytoplasmic PABPC1, which typically binds the polyA track of mature mRNAs in the cytoplasm and is required for translation (35).

**Figure 1:**
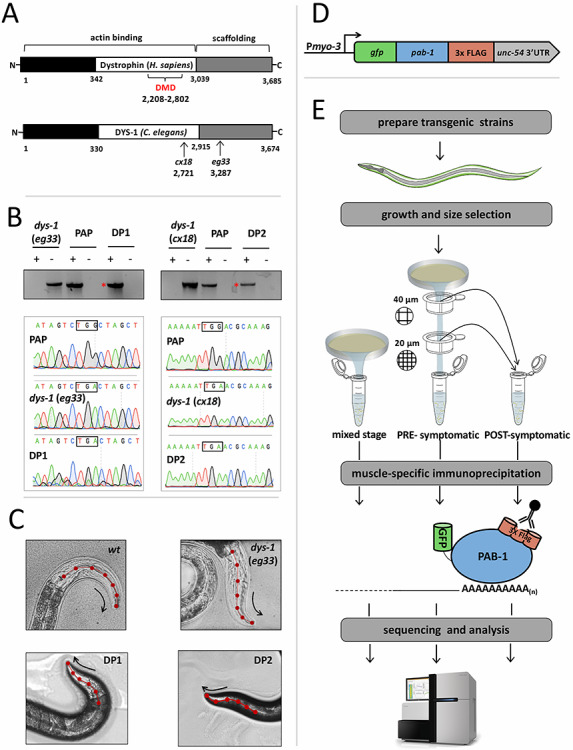
*C. elegans* as a model system to study human DMD. (**A**) Diagram of the functional protein domains in human dystrophin and in the *C. elegans* ortholog *dys-1* (not to scale). Mutational hotspot for DMD mutations in humans is labeled in red. The mutant strains *dys-1*(*cx18*) and *dys-1*(*eg33*) have nonsense mutations in the DMD ortholog *dys-1* (arrows). (**B**) DP1 and DP2 strains retain *cx18* and *eg33* mutations after being crossed with our *wt* PAP strain. Top panel: PCR analysis from genomic DNA extracted from *dys-1*(*eg33*), *dys-1*(*cx18*), DP1, DP2, and *wt* PAP strains. ‘Plus’ denotes primers pairs that confirmed the presence of a single copy integrated construct, and ‘minus’ denotes primers pairs that confirmed the absence of an integrated PAP construct (red asterisks mark the presence of the integrated construct). Bottom panel: trace files produced from the sequencing of the *dys-1* locus confirmed the presence of the nonsense mutation in both DP1 and DP2 strains (black squares). (**C**) *dys-1* strains *dys-1(cx18) and dys-1(eg33)* exhibit a head bending phenotype. *wt* head bending coincides with direction of movement (black arrows), while *dys-1* head bending opposes direction of movement. This head-bending phenotype is retained in DP1 and DP2 strains after crossing the *dys-1*(*eg33*) and *dys-1*(*cx18*) strains with the *wt* PAP strain. (**D**) Diagram of PAP cassette. (**E**) Pipeline used to isolate PRE and POST symptomatic muscle-specific transcriptomes.

Because *dys-1*(*cx18*) and *dys-1*(*eg33*) strains possess nonsense mutations in different portions of the gene (Fig. 1A), we decided to cross our worm strain *myo-3p*::GFP::*pab-1*::3xFLAG (*wt* PAP) with both of these strains (Fig. 1B). The two new strains were named DP1 (*dys-1*(*eg33*;PAP) and DP2 (*dys-1*(*cx18*)*;*PAP). To confirm the presence of the nonsense mutations in the *dys-1* gene in the crossed F1 worms that were GFP-positive in the muscle, we sequenced these mutations using Sanger sequencing (Fig. 1B). We also used a PCR approach to confirm the genomic integration of the PAP construct in the MosSCI locus (36) in both DP1 and DP2 strains (Fig. 1B). In order to further validate our cross, we subjected these worm strains to a Kaplan–Meier survival analysis (Supplementary Material, Figure S1). The average lifespan of N2 worms in this experiment is approximately 21 days, and both new strains behave similarly to their reciprocal pre-crossed strain, with their lifespan drastically declining at day 18, an overall increase in lethality when compared with *wt* worms (Supplementary Material, Figure S1). These two new strains also retain the head-bending phenotype (Fig. 1C). Taken together these results suggest that we successfully crossed both dystrophin-deficient strains with our *wt* PAP strain and that the DP1 and DP2 strains reflect the phenotypes already characterized in the literature before and after our crosses, suggesting our PAP construct did not interfere with the *dys-1* phenotype.

**Figure 2:**
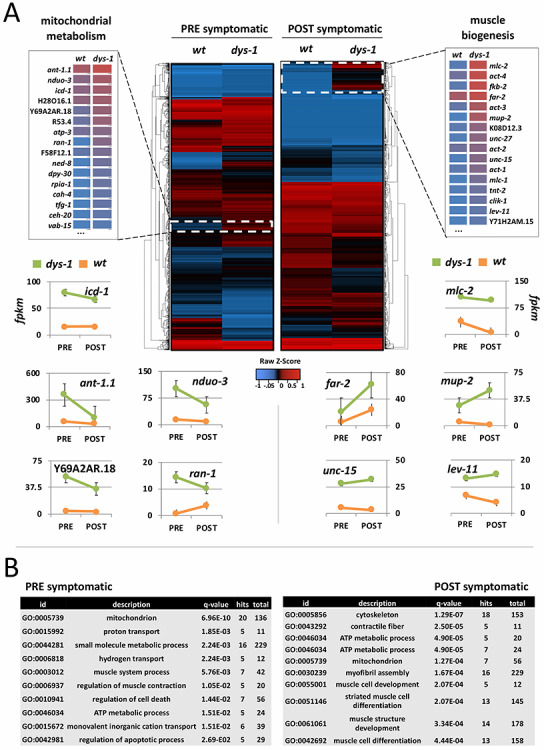
Summary of the muscle-specific PAT-Seq results. (**A**) Heat map summarizing the average change in gene expression of DP1 and DP2 strains as compared to the *wt* PAP strain. White boxes in the heat map mark genes that were upregulated in DP1 and DP2 strains in PRE and POST datasets. The framed gene-lists highlight the change in expression level for selected genes based on function (mitochondrial metabolism or muscle biogenesis) and their rank in our datasets. Below the heat maps, the change in expression level (FPKM) between PRE and POST symptomatic stages of selected genes are graphed linearly (green: median between DP1 and DP2; orange: wild type). ‘*wt’* refers to *wt* PAP, ‘*dys-1’* refers to an average of DP1 and DP2 scores (see Materials and Methods). (**B**) GO term analysis of the top 50 genes uniquely present either in our PRE or POST datasets showed enrichment in genes involved in mitochondria metabolism (PRE) and muscle biogenesis (POST).

We next performed the PAT-Seq experiments, isolating and sequencing muscle-specific mRNAs from DP1 and DP2 strains. We wanted to detect precise changes in gene expression, not only in later developmental stages but also before the onset of the disease was initiated, to have a more comprehensive overview of the dynamic gene changes occurring during the initiation and the progression of the disease. In addition, we wanted to reduce our sample number to simplify the data analysis and increase the depth of our sequencing results. Therefore, we grew large mixed populations of our DP1, DP2 and control *wt* PAP and *dys-1* strains, and then performed sequential mechanical filtrations by size, which led to the isolation of two pools of worms for each strain: one containing embryo-L2 worms, which we labeled ‘pre-symptomatic’ (PRE), and another containing L3-adult worms, which we labeled ‘post-symptomatic’ (POST) (Fig. 1D and E).

We immunoprecipitated, sequenced and analyzed mRNA from mixed stage populations, PRE and POST symptomatic populations from DP1 and DP2 mutant strains, and PRE and POST symptomatic populations from our *wt* PAP strain (16 immunoprecipitations total, see Materials and Methods).

We obtained approximately 900 M reads across all our sequenced samples, with approximately 50 M reads for each dataset (Supplementary Material, Table S1). Within these samples, we were able to map an average of 42% of all the obtained reads to the *C. elegans* genome (WS250) (Supplementary Material, Table S1). Both experimental and biological replicates in each dataset correlate well with each other (Supplementary Material, Figure S2), with ~2000 shared protein-coding genes within each group (Supplementary Material, Table S2).

Our sequencing efforts detected 3747 genes across all datasets (Supplementary Material, Table S3). Within these datasets, we were able to identify uniquely expressed genes in either PRE or POST datasets, or genes with an increase or decrease of at least 2-folds when compared with their respective negative controls (Supplementary Material, Table S4).

About 47% of the total genes identified in this study have been previously mapped by our group in the *C. elegans* body muscle tissue (32) (Supplementary Material, Figure S3A). When we repeated this analysis separately in either our PRE or POST datasets, this percentage increases to ~60% similarity, with >85% identity in our top 250 genes in all our datasets (Supplementary Material, Figure S3A and B).

### Mitochondrial response is implicated in the initiation of symptoms in our PRE datasets

Our PRE symptomatic dataset is primarily composed of embryo to L2 worms. We obtained 1931 protein-coding genes expressed in this group, with 173 unique genes not expressed elsewhere (Fig. 2A and Supplementary Material, Table S3). Within this group, 957 genes have a human ortholog (49.5%) (37). In order to identify changes in gene expression that occur in a *dys-1* background early in development, we compared our DP1 and DP2 PRE symptomatic datasets to corresponding *wt* PAP PRE symptomatic datasets. There are very few genes that change drastically in expression level between the *wt* PAP control and PRE dataset (Fig. 2A and Supplementary Material, Tables S3 and S4). A GO term analysis on the top 50 genes identified enrichment in genes involved in nematode larval development and locomotion, such as *cah-4*, *dpy-30*, *ran-1*, *ceh-20*, *vab-15*, *ned-8*, *rpia-1*, *uri-1*, *tfg-1* and *bus-8* (Fig. 2B).

We detected an unusual abundance of mitochondrial genes involved in ATP production and regulation of apoptotic processes within the top hits identified in our DP1 and DP2 PRE datasets when compared with *wt* PAP PRE datasets (Fig. 2A). *ant-1.1* is an ADP/ATP translocase with a three-fold increase over the median between DP1 and DP2 datasets (when compared with the wild type PRE dataset). This mitochondrial membrane receptor is responsible for transporting ATP synthesized from oxidative phosphorylation into the cytoplasm and absorbs back ADP in a 1:1 molar ratio (38, 39).

In addition to this gene, we also detected gene expression changes in the genes Y69A2AR.18 (5-fold), F58F12.1 (3-fold), *icd-1* (2.5-fold), H28O16.1 (2-fold), *atp-3* (2-fold), all of which are mitochondrial ATP synthase subunits involved in the synthesis of ATP from ADP and inorganic phosphate (Supplementary Material, Table S3).

Approximately 200 genes are instead downregulated in both our DP1 and DP2 PRE datasets (Supplementary Material, Table S3). Many of these genes are also mitochondrial genes, including *Icl-1*, *nduo-4*, *nduo-5*, *cytb-5.2*, *mev-1*, *rad-8*, *atp-4* and Y82E9BR.3 (Supplementary Material, Figure S4 and Supplementary Material, Tables S3 and S4).

Importantly, our approach detected only 173 genes present in the PRE dataset and absent in its *wt* PRE control dataset (Supplementary Material, Table S3). About 43% of these genes have a human homolog (37). These genes most likely reflect the initiation of the disease, but most of them have an unknown function. When aligned to the human proteome, many of them show significant matches to known human genes. *Smo-1* is among those significantly abundant. *Smo-1* is the *C. elegans* homolog of SUMO, a small ubiquitin-like signaling modifier that is attached to proteins dictating localization and function.

### Mitochondrial abundance is disrupted in the absence of functional dystrophin

**Figure 3:**
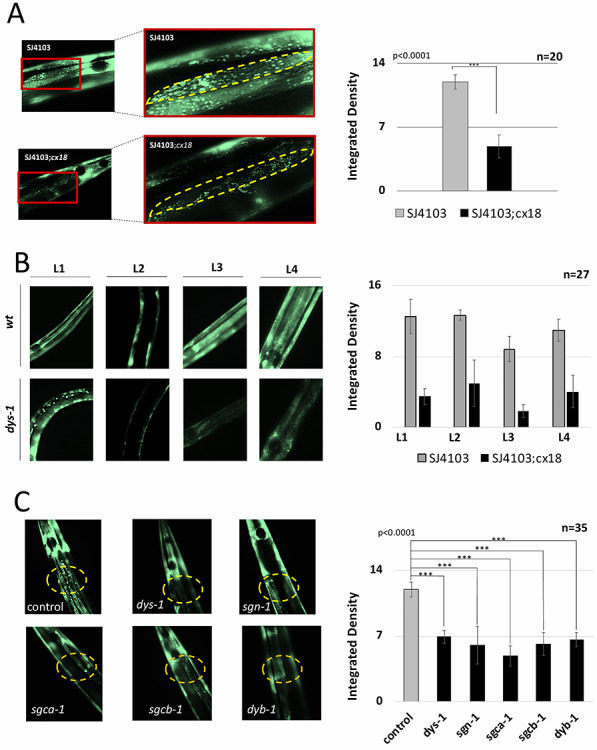
Loss of DYS-1 induces decreased mitochondrial localization throughout development. (**A**) Left panel: body muscle specific mitochondrial fluorescence in SJ4103 strains in *wt* and *dys-1*(*cx18)* genetic backgrounds. Enlargements expand the single muscle cell at the base of the pharynx (yellow). Right panel: quantification of the average brightness (integrated density) of body muscle specific mitochondria in SJ4103 strains in *wt* and *dys-1(cx18)* genetic backgrounds. (**B**) Left panel: body muscle specific mitochondrial fluorescence in each developmental stage decreases in the *dys-1(cx18)* strain. Right panel: quantification of the relative brightness. *n* = 27 (**C**) Known members of the DGC were knocked down using RNAi in SJ4103 strains, and brightness of mitochondrial fluorescence was quantified as average integrated density using ImageJ analysis. Representative images for each gene knockdown are pictured in the left panel and the quantification is displayed in the right panel (*P* < 0.0001 *t*-test).

We were intrigued by the presence of a large number of genes involved in apoptosis and mitochondria metabolism in both our DP1 and DP2 PRE and POST datasets as compared with their corresponding *wt* PRE and POST datasets, and decided to further study and validate these findings using a different genetic approach. We crossed the *dys-1*(*cx18*) strains with the SJ4103 strain, which restricts the expression of GFP to mitochondria of body muscle tissue (40) and used the resultant new strain to study if the loss of *dys-1* alters the abundance of muscle mitochondria throughout development. This new strain (SJ4103;*cx18*) is still hyperactive, presents the same head bending phenotype observed in the *dys-1*(*cx18*) and displays significantly less mitochondria localized in the body muscle cells (Fig. 3A). Loss of mitochondria in the body wall muscle was also detected throughout development (Fig. 3B). Importantly, the loss of muscle mitochondria is DGC-dependent, as it can be recapitulated through the silencing of *dys-1* or other members of the dystrophin complex using RNA interference (Fig. 3C).

### Genes involved in myogenesis and muscle contraction are abundant in the POST dataset

In order to identify trends in gene expression that occur later in development in a *dys-1* background, we compared our DP1 and DP2 POST datasets to the corresponding *wt* POST datasets. In our POST dataset, we detected 2273 protein-coding genes across both DP1 and DP2 strains. Within this group, 1148 genes have a human ortholog (50%) (37). Only 58% of genes are shared with the PRE dataset (Fig. 2A and Supplementary Material, Table S3). This discrepancy is probably consistent with the progressive nature of the disease, and the associated destructive changes in muscle structure and overall health.

Essential constituents of the mitochondrial metabolism are also overexpressed at this stage, although at lower levels than in our PRE dataset. They include the mitochondria ATP synthase *Icd-1*, *ant-1.1*, H28O16.1, Y69A2AR.18, *atp-2* and *atp-3*, *nduo-1* (NADH-ubiquinone oxidoreductase), W09C5.8 (cytochrome c oxidase) (Fig. 2A and Supplementary Material, Tables S3 and S4).

The most abundant class of genes in this stage are involved in myogenesis. This includes *mlc-1* and *mlc-2* (myosin light chain), *mup-2* (troponin), *act-1* (actin), *unc-27* (troponin), *lev-11* (tropomyosin) and *unc-15* (paramyosin) (Fig. 2A and Supplementary Material, Tables S3 and S4). This result is consistent with muscle hypertrophy or active replacement of muscle in humans and is in line with previous studies in worms, which show marked hypertrophy and increased myofibril diameter following high intensity muscle exertion through burrowing (41). In this context, the increase in transcription we observed may suggest a hidden compensatory signaling pathway activated in the absence of *dys-1* that precedes cell death. We chose to explore this trend in our sequencing results in detail, as changes in muscle structure have been reported as the mostly likely contributor to the *dys-1* phenotype in surrounding literature (42). Previous works have shown that interactions between DYS-1 and other structural proteins at the dense body including DYC-1 are essential in muscle adhesion and structure, and severing this interaction potentially leads to degradation of the sarcomere and muscle structure (42). Of note, when we overexpressed a GFP-tagged version of the *C. elegans* homolog dystrobrevin (*dyb-1*) in *dys-1*(*cx18*), we detected an intact DGC in the body muscle in our POST symptomatic worms (Supplementary Material, Figure S5), suggesting that in *C. elegans*, the presence of a full length DYS-1 protein may be dispensable for the assembly of the DGC.

Collectively, ~250 genes were significantly downregulated in our DP1 and DP2 POST datasets. *Unc-22* encodes ‘twitchin,’ a protein required for proper assembly of thick filaments into A-bands, and regulation of myosin activity (43, 44) and is 2-times less abundant in our POST datasets when compared with our negative control. B0336.3, an ortholog of human RBM26/27 involved in body morphogenesis and striated muscle myosin thick filament assembly, is three-times less abundant in our POST datasets when compared with our negative control. The muscle genes *ins-14*, *aex-5* and *ttn-1* were also found downregulated in this analysis (Supplementary Material, Tables S3 and S4).

Only 38 genes are uniquely present in our POST dataset. The majority of these genes have unknown functions (Supplementary Material, Table S3). About 11 of these genes (29%) have a human ortholog (37).

### Aberrant splicing events are pervasive in the POST symptomatic *dys-1(eg33)* strain

A recent study identified alternative splicing defects in transcriptomes from myotonic dystrophy skeletal muscle and heart (44). To investigate whether this phenomenon may be present in DMD as well, we first examined our *dys-1(eg33)* POST datasets for changes in expression of genes related to splicing. Several SR proteins (*SRP-1*, *SRP-2*, *SRP-3* and *SRP-5*) and snRNPs (*SNR-1*, *SNR-2*, *SNR-5* and *SNR-6*) showed at least 2-fold increase or decrease in expression levels in our PRE and POST datasets, when compared with their respective wild type controls (Supplementary Material, Figure S6A). SR proteins and snRNPs are splicing factors involved in both constitutive and alternative RNA splicing and function in a dosage-dependent manner, meaning that their relative abundance dictates different splicing events (45). Because the expression levels of these genes were particularly altered in our PRE and POST datasets, we decided to test if defects in RNA splicing were also present in these stages. For this analysis, we focused on our *dys-1*(eg33) strain.

We mapped 26 692 changes in splicing junction usage in this mutant strain in both our PRE and POST muscle transcriptomes when compared with our PRE and POST control datasets, respectively (Supplementary Material, Figure S6B). Within this pool we detected extensive differences in splice junction usage in our DP1 strain (Supplementary Material, Figure S6B and C). About 10% of these identified RNA splicing junctions (1413) used novel acceptors and donors, and many of these occur with more than 2-fold-change in our PRE and POST datasets.

### Transcriptomes of *dys-1(eg33)* and *dys-1*(*cx18*) are similar but not identical

We then decided to study the variability in gene expression present between the two *dys-1* strains. A recent study (19) found that the *dys-1*(*eg33*) strain is more clinically relevant than *dys-1*(*cx18*) for muscular dystrophy studies in *C. elegans*. The *dys-1*(*eg33*) strain is weaker than *dys-1*(*cx18*) strain and its wild-type counterparts. This mutant strain exhibits impaired thrashing in liquid and strong mitochondrial network fragmentation in the body wall muscles. Although the molecular mechanisms responsible for these phenotypic differences are not yet known, it is possible that the difference in location of the mutation in the *dys-1* gene could be a contributor. These differences could be explained by the presence of different genetic programs executed in the muscles of these two strains driven by specific protein domains which are present in the *dys-1*(*eg33*) mutant strain and lost in the *dys-1*(*cx18*) mutant strain. Our transcriptome results in DP1 and DP2 strains and their two biological replicates support this hypothesis. Although they are very similar, the presence and the expression levels of the genes we mapped in these two mutant strains revealed subtle differences (Supplementary Material, Figures S3C, S4, S6 and Supplementary Material, Table S4).

**Figure 4:**
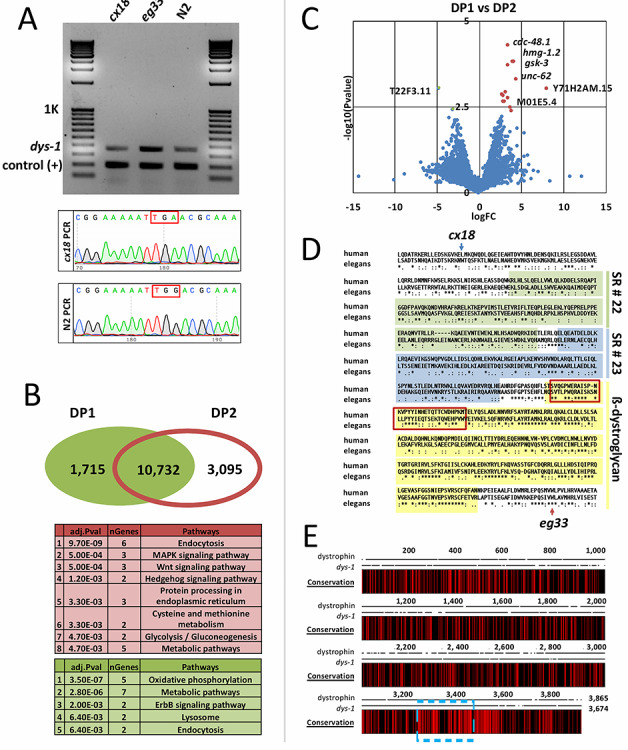
*dys-1 is expressed in dys-1*(*eg33*) and *dys-1*(*cx18*) strains. (**A**) Top panel: Detection of *dys-1* transcript *in dys-1*(*eg33*) and *dys-1*(*cx18*) mutant. RT-PCR analysis was performed on total RNA using primers that anneal on either side of the *cx18* mutation in the *dys-1* transcript, mixed with primers which anneal to the *myo-2* gene within exons 5 and 6 (control +). Bottom panel: the amplicon corresponding to the bands observed in the *dys-1(cx18)* and N2 lanes in the top panel were purified and sequenced using primers surrounding the region containing the *cx18* mutation. The red box shows the location of the nonsense mutation in *dys-1*(*cx18*) mutant. (**B**) Top panel: Venn diagram comparing the number of genes detected in DP1 and DP2 strains in their respective POST datasets. Bottom panel: top list of a GO Term analysis performed using the unique genes detected in the DP1 or DP2 strains (**C**) Volcano plot depicting gene expression changes in common genes between DP1 and DP2 strains. Significantly overexpressed genes in the DP1 strain are shown as a red dot (*P* ≤ 0.005). (**D**) Alignment of the human DMD gene and *C. elegans* ortholog *dys-1* with conserved functional domains highlighted. The WW domain is outlined in red. The two mutations *eg33* and *cx18* are marked with an arrow. (**E**) Map of sequence conservation between human and *C. elegans* orthologs of the dystrophin gene with the β-dystroglycan binding domain outlined in blue.

We decided to test if *dys-1* mRNA was indeed present in the muscle tissue of these two strains and is perhaps responsible for the phenotypic differences detected between these two mutant strains. Using an RT-PCR approach, we consistently detected the presence of the *dys-1* transcript in both *dys-1*(*cx18*) and *dys-1(eg33*) strains, with no obvious degradation, suggesting that at least at mRNA level, these two transcripts are stably present in dystrophic muscle (Fig. 4A). We then compared the gene population shared between our DP1 and DP2 mutant strains in the POST datasets. As expected, the majority of genes are shared between both datasets, but we also detected unique gene populations expressed only in our DP1 and DP2 strains (Fig. 4B and Supplementary Material, Figure S4). Approximately 30% of the total genes identified in DP2 dataset (*dys-1(eg33)*) were not detected in our DP1 (*dys-1(eg33)*) mutant strain (Fig. 4B). Most of these genes are involved in signaling pathways, such as the MAPK and the Wnt pathways, which are known to be involved in muscle formation (46). Some of the genes, including *gsk-3* and *unc-62* were also detected in the DP1 (*dys-1(eg33)*) strain but show a striking increase in fold change in the DP2 (*dys-1(cx18)*) strain (Fig. 4C).

We then aligned the protein sequences of human dystrophin and worm *dys-1* and mapped the location of the *dys-1* mutation in both *dys-1*(*eg33*) and *dys-1*(*cx18*) strains. We found that the last two spectrin-like (SR) repeats, the WW domain and the β-dystroglycan binding domain are present in the *dys-1*(*eg33*) strain but missing in the *dys-1*(*cx18*) (Fig. 4D). These elements are critical for binding β-dystroglycan, and in humans anchor the dystrophin complex to the sarcolemma. This portion of the protein is overall the most conserved region between the human dystrophin and DYS-1 (Fig. 4E).

### A novel synthetic screen to identify genetically linked *dys-1* targets

Our muscle-specific transcriptome approach identified specific genes differentially regulated in both dystrophin-deficient samples across all replicates. Among several trends, we mapped an enrichment of genes involved in mitochondrial metabolism in our PRE dataset and genes involved in the establishment and maintenance of muscle structure and function in our POST dataset*.*

In order to validate and expand the biological significance of these results, we decided to perform a targeted RNAi knockdown genetic screen to test genes identified in our study for their ability to enhance muscle damage in dystrophin-deficient strains. We reasoned that if there were genetic compensatory mechanisms to increase gene expression, perhaps to counteract decline in muscle structure and function, the knockdown of these upregulated genes detected in our screen would lead to an increase of the impairment of muscle viability that would, in turn, suggest a genetic link between *dys-1* and the tested genes.

Unfortunately, the *dys-1* phenotype is subtle when compared with *wt* worms. For this reason, we decided to use a previously established *dys-1* mutant strain *dys-1(cx18); hlh-1(cc561ts)* that uses a background mutation to enhance *dys-1* symptoms to create a more definitive and scorable phenotype.

It has been previously shown that combining dystrophin mutations with mutations in the transcription factor MyoD exacerbates dystrophic phenotypes in mice and that this combination has a similar effect in *dys-1* strains (47). The temperature-sensitive strain *hlh-1(cc561ts)* contains a hypomorphic mutation in the *C. elegans* homolog of MyoD, *hlh-1*, which renders these strains viable at the permissive temperature 15°C and severely uncoordinated with defects in body muscle morphology at non-permissive temperatures above 20°C (48). This strain has been already crossed with *dys-1*(*cx18*), producing a new strain named *dys-1(cx18); hlh-1(cc561ts)*, which showed a similar phenotype at non-permissive temperature.

**Figure 5:**
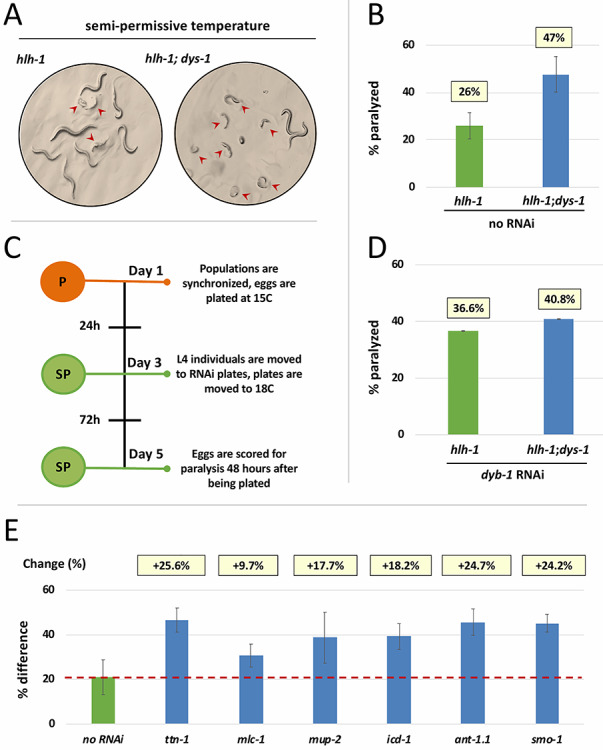
A synthetic screen to identify genetically linked *dys-1* targets (**A**) Single (*hlh-1*) or double (*hlh-1; dys-1*) mutants scored for paralysis at semi-permissive temperature. Red arrows indicate worms that were scored as paralyzed. (**B**) Quantification and comparison of the average incidence of paralysis for single versus double mutant strains at semi-permissive temperature. The values above each bar chart represent the average score between all replicate plates for each respective strain (*n* = 543). (**C**) Overview of the semi permissive RNAi screen protocol. P, permissive temperature (orange); SP, semi permissive temperature (green). (**D**) *dyb-1* knock-down increased incidence of paralysis in single mutants. Percentage of paralyzed worms following RNAi for *dyb-1,* with average percentage of paralysis of five replicate plates summarized above each bar (*n* = 385). (**E**) Synthetic paralysis RNAi assay to selected test genes found to be upregulated in this study. Each gene chosen for knockdown is displayed as the difference in paralysis observed between the *hlh-1* and *hlh-1;dys-1*(*cx18*) mutants and normalized to the difference of paralysis obtained from semi-permissive control screens described in B (21%). Red dotted line marks the point of normalization for change in paralysis. The change in incidence of paralysis compared to the difference without RNAi (B) is shown on top of each bar.

In order to ameliorate the uncoordinated defects and lethality obtained at 20°C, we decided to decrease this temperature to 18°C (semi-permissive). At this temperature, ~75% of the single mutants, and ~50% of the double mutants are still viable (Fig. 5A and B).

Populations were synchronized and grown at 15°C (permissive) until they reached the L4 stage and were then moved to a semi-permissive temperature of 18°C and allowed to lay eggs for 24 h (Fig. 5C). At semi-permissive temperature, the single mutant strains *hlh-1* gave rise to the progeny of which 26% developed with severe defects in body morphology and were almost completely paralyzed (Fig. 5B). Under the same conditions, the double mutant strains *hlh-1*; *dys-1* yielded a 47% of paralysis (21% increase), and the hatched larvae shown severe defects in body morphology (Fig. 5A and B).

We then selected a subset of representative upregulated genes identified by PAT-Seq (indicated with an asterisk in Supplementary Material, Table S3). These genes were also chosen based on function, primarily because of their known roles in mitochondrial metabolism, muscle structure and signaling function. We speculated that if these genes were indeed abundant in symptomatic *dys-1* worms as a compensatory mechanism to counteract paralysis, their depletion in the double mutant background would enhance paralysis when compared with the single mutant background.

The RNAi experiments were performed at the semi-permissive temperature of 18°C. Worms were scored for the incidence of morphological defects and resulting paralysis (Fig. 5C). The results between control and experimental strains were compared with each other, and then to the observed differences in semi-permissive experiments performed on single and double mutant strains in the absence of RNAi. As a control, we initially performed a knockdown of *dyb-1*, a known member of the nematode DGC (22). We reasoned that *dyb-1* RNAi in the single *hlh-1* mutant background should increase the rate of paralysis but have no effect in the double mutant strain, because in this strain *dys-1* function is absent and its interaction with *dyb-1* has been already severed.

As expected, the knockdown of *dyb-1* was able to increase the incidence of paralysis in the single mutant, which was comparable with the result obtained with the double mutant strain in semi permissive control experiments (Fig. 5D).

We then tested select genes from our muscle and signaling-related gene pools. Although some of the genes were synthetic lethal in combination with the *hlh-1* background mutation (*act-1*, *cmd-1*, *unc-27* and *unc-15*), and could not be tested further, the majority of genes tested were able to enhance paralysis, although to differing degrees (Fig. 5E and Supplementary Material, Table S3).

## Discussion

Here, we have used the nematode *C. elegans* to study the cell-autonomous molecular events associated with the functional loss of the dystrophin gene, which leads to DMD in humans. We have performed genetic crosses to establish new transgenic strains that serve both as a model for DMD, and as a functional tool to perform high quality, muscle-specific RNA-IPs at a resolution that has not yet been achieved. Using these strains, we have isolated, sequenced and analyzed muscle-specific transcriptomes during disease progression and identified several differentially regulated pathways in the dystrophic nematode muscle.

We have also developed a novel, temperature-based genetic screen and performed a quantitative analysis to assess the functional significance of the dysregulated genes identified in our sequencing results, thus confirming their potential contribution towards DMD initiation and progression. This approach will further allow a direct evaluation of the role of specific genetic pathways in the clinical severity of DMD, which will be fruitful in developing drug targets for treating DMD patients. Our dystrophin-deficient strains DP1 and DP2 display altered molecular pathways that are very similar to those observed in *mdx* mice and DMD patients, strongly implying that this model system phenocopies many aspects of the disease at the molecular level (49–51).

These new strains recapitulate phenotypes previously characterized in the literature for *dys-1*(*cx18*) and *dys-1*(*eg33*), with shortened average and maximum lifespan, hyperactivity and excessive bending of the head (21, 25) (Fig. 1C and Supplementary Material, Figure S1), suggesting that introducing the PAP cassette into the *dys-1* genetic background has not altered the *dys-1* phenotype.

The longevity curves obtained with our lifespan assay were somewhat shorter that those initially reported by Oh and Kim (2013) (25). In their experiments, animals are placed on nematode growth media (NGM) plates containing Fluorodeoxyuridine (FUDR) at a concentration of 100 μg/ml, and they observed that the majority of their dystrophic worms survived until the day 20. In our experiments, 40–50% of the tested animals were still alive at the day 20. We have used a different lifespan assay protocol which adds less FUDR in the plates, to a final concentration of 50 μM, and this change could be in part responsible for our observed decrease in survival (52). In addition, our scoring method of lethality could have also accounted for these differences (see Material and Methods). Importantly, we observed consistency in lethality between the *dys-1*(*eg33*) and the *dys-1*(*cx18*) strains before and after the crosses with our *myo-3p*::GFP::*pab-1*::3xFLAG strain, suggesting consistency within our assay.

Our study uncovered ~2000 muscle protein-coding genes with altered expression levels in the early and late-stage dystrophic muscle when compared with wild type muscle tissues. Among this group, ~500 genes showed at least 2-fold increase in both the PRE and the POST dataset, and across both biological replicates for each sample. In order to reduce background signal, we have applied a stringent bioinformatic filter to restrict our analysis to the top 30–40% of the total genes identified in this study (Supplementary Material, Figure S3D), which may have in turn lowered the number of genes considered, but provided us with higher quality results (Supplementary Material, Figure S3D).

Our mapped reads from our POST datasets were consistent with our past studies across both biological replicates (31). Of note, we obtained less than average mapped raw reads in all our PRE datasets (Supplementary Material, Table S1), which may have biased our results. The original PAT-Seq approach was optimized using mixed stage populations as the starting material for the immunoprecipitation steps (31, 32), and it was never applied to isolate RNA from early stages such as embryos and L1/2 worms. The mechanical filtration steps we used to isolate these stages may have also introduced unwanted noise in our PRE dataset, which was recovered from the flow-through of the strainers. The L3/L4 and adult worms in our POST datasets were instead retained by the strainers and isolated from the flow-through.

Although we obtained fewer mapped reads, it is important to note that the total number of unique genes we mapped in our PRE datasets is similar across all datasets (but with less coverage) (Supplementary Material, Table S2), suggesting that the distribution of our mapped reads in our PRE dataset for abundant genes is not biased. In addition, our Principal component analysis (PCA) in Supplementary Material Figure S3C shows that the genes and their abundance in both replicates in our PRE datasets are very similar to each other, also suggesting consistency. Of note, when compared with the top 250 genes identified in our past muscle-specific transcriptome from mixed stage worms (32), genes mapped in both our PRE datasets show a similar trend to those mapped in our POST datasets (Supplementary Material, Figure S3B), further suggesting that the genes identified in our PRE dataset are not random, but are the direct result of our immunoprecipitation approach.

Despite the differences in mapped reads between PRE and POST datasets after filtration, the reads obtained are not likely to have been biased by the size selection method itself. It has been previously shown that although there are measurable differences in strength between wild-type and *dys-1(eg33)* and *dys-1(cx18)*, which cannot be attributed to differences in worm diameter (19), and our filtration control experiment in Supplementary Material, Figure S1B confirmed that there are no significant differences in stage enrichment in PRE and POST populations in the *dys-1* strains (Supplementary Material, Figure S1B).

Our study revealed two distinct sets of genes that contribute to the DMD phenotype in *C. elegans*. The first set is primarily activated early in development and is composed of genes involved in mitochondrial homeostasis, cell death and protein degradation signaling in the muscle. The second set is activated during the final half of the developmental cycle, continues through adulthood, and is involved in the establishment and maintenance of muscle structure.

There are currently a number of studies in *mdx* mice reporting the involvement of both pathways in DMD progression (18, 21, 53), but prior to this study it was unclear how these two pathways were intertwined with each other, and in what order these events occurred. This question is important, as impaired mitochondrial metabolism has already been observed in *mdx* myoblasts, in which a functional DGC has not yet been assembled (54).

Our results suggest that these two pathways do not occur simultaneously; the mitochondrial dysfunction is detected early in disease progression, in accordance with previous findings (18), either before symptoms are initiated or while they are still mild.

In the PRE dataset, we detected the differential regulation of numerous mitochondrial genes associated with the glycolysis pathway and ATP/ADP transport such as *ant-1.1*, *nduo-2*, *nduo-3* and *atp-3* (Fig. 2 and Supplementary Material, Table S5). Proteomics studies in *mdx* mice also support these results (18). Muscle contraction is dependent upon ATP production and usage, and its abundance is tightly regulated in healthy muscles.

Aberrant mitochondrial activity has been previously reported in DMD patients and animal models of DMD (17, 19, 55–58), even prior to dystrophin assembly at the sarcolemma (54). Furthermore, previous studies in *C. elegans* have characterized the functional relationship between DYS-1 and surrounding members of the DGC and have found that *dys-1* mutations lead to the mislocalization of the Ca^2+^-gated K^+^ channel protein SLO-1 resulting in altered intracellular calcium levels (59). We also detect a decrease of mitochondrial localization in the muscle of *dys-1* worms (Fig. 3), which is consistent with these results. Our RNAi experiments in Figure 3C also confirmed the importance of the DGC in relation to mitochondrial localization, because the selective knockdown of four members of this complex caused a decrease of mitochondrial localization to the body muscle. Taken together, our results validate these studies and highlight an important signaling role for *dys-1*, which evidently functions outside of its accepted role as a scaffolding protein in the DGC.

After the onset of symptoms in post-symptomatic samples, while mitochondrial dysfunction is still present (Fig. 3), we detect a second pathway, which perhaps tries to actively compensate for the loss of muscle structure by overexpressing genes involved in muscle formation. We do not know if these transcripts are indeed carrying out a compensatory mechanism, and more experiments need to be performed to further validate this finding. Of note, many of them were able to enhance paralysis in our RNAi experiments in Figure 5E, suggesting that they are functionally translated.

Our results have shown that *dys-1*(*eg33*) and *dys-1*(*cx18*) transcriptomes, while highly similar, are not identical (Fig. 4B, C and Supplementary Material, Figures S3C, S4). A previous study found *dys-1*(*eg33*) mutants to be significantly weaker than *dys-1*(*cx18*) and their wild-type counterparts (19), but the molecular mechanisms driving these differences was not known. *Dys-1*(*eg33*) possess a weaker thrashing score in liquid than *dys-1*(*cx18*) (19) and display stronger mitochondrial network fragmentation in the body wall muscles (19). *dys-1*(*eg33*), but not *dys-1*(*cx18*), also responds to prednisone and melatonin, showing improved muscular strength, thrashing rate and mitochondrial network integrity in response to treatment with these compounds (19). Our results align with these findings, as we were able to show that *dys-1* mRNA is surprisingly transcribed in both strains with little detectable degradation (Fig. 4A), suggesting that two different DYS-1 truncated proteins are present in these two strains, and potentially act as hypomorphic alleles. We do not yet know why the nonsense mutations in the *dys-1* genes are not recognized and degraded by the nonsense mediated decay pathway.

The shorter *dys-1*(*cx18*) strain lacks both the DYS-1 WW domain and the β-dystroglycan binding domain, which in the human dystrophin gene are responsible for binding to β-dystroglycan and anchoring the protein to the sarcolemma. The lack of this element is perhaps able to induce the altered signaling pathways we have detected in the *dys-1(cx18)* strain, which in turn may be responsible for the differences in both phenotype and gene expression observed between these two strains. Alternatively, the presence of this element followed by a truncated C-terminal domain may induce muscle stress in burrowing experiments and allows for drug sensitivity. In support of this hypothesis, we detected a strong conservation in this region between the human and the worm dystrophin ortholog (Fig. 4D), which implies there is a lost functional role in the *dys-1*(*cx18*) strain. More experiments need to be performed to address this issue. Importantly, DGN-1, the worm ortholog of the β-dystroglycan gene, has been found to be located outside the muscle and does not bind DYS-1 (60). If correct, the loss of the β-dystroglycan binding site in the *dys-1*(*cx18*) mutant strain cannot be directly responsible for the functional differences observed between the *dys-1*(*cx18*) and the *dys-1*(*eg33*) strains. However, in contrast with this past study, we have repeatedly identified *dgn-1* in our *C. elegans* muscle transcriptomes in this study and elsewhere (31, 32), suggesting that perhaps there could be a small population of DGN-1 in muscle which is responsible for anchoring *dys-1* to the sarcolemma. This issue requires further studies to closely characterize the role of these two truncated versions of DYS-1 and how they may impact cellular signaling in the muscle.

Our results in Supplementary Material, Figure S6 found widespread splicing disorders in both our PRE and POST datasets. Our study identified thousands of aberrant splice junctions in the *dys-1*(*eg33*) mutant strain, which correlates with altered abundances of RNA splicing factors such as snRNPs and SR proteins. Disorders in mRNA splicing have also been observed in 120 transcriptomes from skeletal and heart muscle derived from healthy and dystrophic biopsies and autopsies (44). In this context, *C. elegans* again phenocopy these defects and provides a more robust platform to study the genetic mechanisms caused by these lesions in the context of DMD. We do not know if the widespread aberrant splicing we detected is able to escape NMD or it is subjected to it. Of note, in our muscle datasets we scarcely detected genes involved in this process, suggesting that perhaps, if NMD occurs is not able to completely prevent their translation.

Our RNAi experiments in Figure 5 most effectively screen for genes that play a role in the muscle and work cooperatively with dystrophin beginning early in development. The effects of the RNAi knockdown are scored in the first hours after L1 animals hatch. In this light, the results of our semi permissive control experiments showed that the absence of dystrophin was able to affect the very early development of the embryos that ultimately hatched with severe muscle defects. This finding coincides with our sequencing results in our PRE datasets, which suggested dystrophin may play an early role, both in development and in regulating mitochondrial function, and is not simply a structural protein whose absence affects developed muscle after continuous contraction.

The changes in gene expression observed in our POST dataset suggest that many of the genes encoding structural proteins of the sarcomere are involved in the progression of dystrophin-induced muscle damage (Fig. 2). Our RNAi experiments in Figure 5 confirmed that these genes act in a *dys-1* dependent manner and verify their role in a compensatory mechanism that may allow *C. elegans* to increase transcription of muscle-structure related genes in response to muscle damage.

*mup-2*, a gene that codes for the ortholog of muscle protein troponin, TTN-1 a titin-like protein, and *mlc-1*, an ortholog of myosin regulatory light chain were all able to increase the incidence of muscle defects and paralysis in the dystrophin-deficient strain *dys-1(cx18); hlh-1(cc561ts)*, without inducing the same effect on the control strain *hlh-1(cc561ts)* (Fig. 5E).

Taken together these results suggest that *mup-2, ttn-1* and *mlc-1* are all genetically connected to *dys-1* and are not only overexpressed as transcripts when visible symptoms begin, but are also expressed as proteins, as their dosage is necessary to increase paralysis in a *dys-1* dependent manner.

Outside this group of genes selected because of their role in muscle structure, we also studied upregulated genes essential in several signaling pathways. *Icd-1* is the β-subunit of the nascent-polypeptide associated complex*. Icd-1* was significantly upregulated in POST symptomatic data sets. It mediates proteins transport to mitochondria (61) and is necessary and sufficient to suppress apoptosis (62). *icd-1* knockdown induces a two-fold increase of incidence of muscle defect and paralysis in *dys-1* dependent manner (Fig. 5E), suggesting that these two genes are also genetically connected.

These synthetic paralysis phenotypic experiments in Figure 5 are challenging to analyze. Unfortunately, *dys-1* mutations do not lead to a drastic muscle phenotype and aggravation of phenotypic severity using the *hlh-1* ts allele is needed in order to simplify scoring. Because *hlh-1* is involved in muscle development, these results may report a developmental arrest phenotype caused by disruption in muscle elongation during embryogenesis. Although this may be the case, our readout is the comparison of the incidence of lethality induced by a given RNAi experiment in worms with and without the *dys-1* mutations. Because of this, the scored variation in morphological defects mirrors the contribution of the loss of *dys-1* to the developmental arrest phenotype, which is induced at a much lower rate by *hlh-1* alone in all our RNAi experiments.

We opted to use this genetic background because it was previously published and successfully used in a similar approach (47). In addition, while some groups have used approaches without modified genetic backgrounds to define impaired movement and muscle function, such as thrashing or tracking of movement, we elected to use this semi permissive assay because it is rapid, reveals readily scorable differences between single and double mutants, and is easily adaptable for future large-scale experimentation.

Another observed limitation in using our approach for RNAi screens was the incidence of synthetic lethality. When combined with background mutations in *hlh-1*, several genes involved in the development of muscle proved to be embryonic lethal when knocked down. Because this synthetic lethality achieved the same penetrance in single and double mutant strains, it prevented the scoring of differences between the two strains. It is important to note that this did not occur in the bulk of our experiments that knocked down muscle-specific genes, meaning it is still feasible to use this method to screen the majority of genes in the genome, both muscle-specific and ubiquitously expressed.

In conclusion, our analysis of dystrophin deficient muscle transcriptomes has confirmed the signaling role of dystrophin in nematode muscle and allowed us to further study the consequences of dystrophin deficiencies in great detail.

## Materials and Methods

### Preparation of nematode transgenic strains

Body muscle-specific PAP expressing transgenic lines (*wt* PAP) were obtained from a previous publication (31). SJ4103 strains were obtained from the CGC, which is funded by NIH Office of Research Infrastructure Programs (P40 OD010440). Young adult *C. elegans* worms were isolated on NGM agar plates seeded with OP50–1 and incubated at 31°C for 3.5 h. To prepare the crosses, plates were then incubated for 4 days at 20°C and males were isolated from populations. Groups of five males were paired with 10 L4 hermaphrodites and incubated for 3 days at 20°C. Crosses between *wt* PAP and *dys-1*(*cx18*) and *dys-1*(*eg33*) strains, and crosses between SJ4103 and *dys-1(cx18)* strains were screened for GFP fluorescence using a Leica DM13000B microscope. Strains positive for fluorescent markers were subjected to Sanger sequencing for the verification of mutations in the dystrophin gene using the following primers: *dys-1*(*cx18*)_F: GGCTTAATATGAGCTGGACGAAG, *dys-1(cx18)*_R: CGCTGTCCATCTTCTTGTGG, *dys-1(eg33)*_F: GGACGGTCATGCGACCC, *dys-1(eg33)*_R: TTTGCACACGTTGCATTTGG. In order to simplify the nomenclature, we have renamed the crossed strain *dys-1(eg33)*/PAP to DP1 and the crossed strain *dys-1(cx18)*/PAP to DP2 throughout this manuscript.

### Preparation of PRE and POST symptomatic PAT-Seq strains for RNA immunoprecipitation

*C. elegans* strains were divided into pre-symptomatic (PRE) and post-symptomatic (POST) pools using mechanical filtration using pluriStrainer cell strainers (pluriSelect). Mixed-stage populations were harvested from NGM agar plates seeded with OP50–1 and pelleted at 1500 rpm. Solid pellets of approximately 2 ml were sequentially pipetted through 40 and 20 μm nylon cell strainers. The collected flow-through contained embryo/L1/L2 population, which we renamed PRE symptomatic. Worms retained by both filters, which included L3/L4/Adult population, were then combined and renamed POST symptomatic. During the filtration process, pellets were continuously resuspended in M9 buffer to prevent worms from being crushed or forced against mesh filters.

### Mechanical filtration control experiments

N2, *dys-1(eg33)*, and *dys-1(cx18)* worm strains were grown on standard NGM plates as mixed-stage populations until 200 μl solid pellets were obtained for each strain. The entirety of this pellet was passed through a 40 μM cell strainer. The pellet was continuously resuspended in M9 during the filtration process. The retained populations and the flow through were both pelleted and resuspended in 500 μl of M9 buffer. From this resuspension, 25 μl samples were taken both from the retained population and the flow through populations and each stage present in this aliquot was scored. About 25 μl samples were taken in triplicate for each worm strain for both retained and flow through samples. Values for each stage were calculated as a percentage of the total number of worms in each 25 μl sample and then averaged across replicates. The results of this analysis are shown in Supplementary Material, Figure S1B.

### RNA immunoprecipitation

*C. elegans* strains used for RNA immunoprecipitations were maintained at 20°C on NGM agar plates seeded with OP50–1. Populations were passaged until a 1 ml pellet for mixed stage IPs and a 2 ml pellet for split stage IPs was obtained following centrifugation at 1500 rpm. Following the isolation of PRE and POST symptomatic populations through mechanical filtration, worm strains were harvested, suspended and crosslinked in 0.5% paraformaldehyde solution for 1 h at 4°C. Worms were then pelleted at 1500 rpm, washed with M9 buffer, and flash-frozen in an ethanol-dry ice bath. Pellets were thawed on ice and suspended in 2 ml of lysis buffer (150 mm NaCl, 25 mm HEPES, pH 7.5, 0.2 mm dithiothreitol (DTT), 30% glycerol, 0.0625% RNAsin, 1% Triton X-100) (31). Lysate was then subjected to sonication for 5 min at 4°C (amplitude 20%, 10 s pulses, 50 s rest between pulses) using a sonicator (Fisher Scientific), and centrifuged at 21 000× *g* for 15 min at 4°C. 1 ml of supernatant was added per 100 μl of Anti-FLAG^®^ M2 Magnetic Beads (Sigma-Aldrich) and incubated overnight at 4°C in a tube rotisserie rotator (Barnstead international). mRNA immunoprecipitations were carried out as previously described (31, 32). Total precipitated RNA was extracted using Direct-zol RNA Miniprep Plus kit (R2070, Zymo Research), suspended in nuclease-free water and quantified with a Nanodrop^®^ 2000c spectrophotometer (Thermo-Fisher Scientific). Each RNA IP was performed in duplicate to produce two biological replicates for the following samples: DP1 PRE, DP2 PRE, DP1 POST, DP2 POST, *wt* PAP PRE and *wt* PAP POST.

### cDNA library preparation and sequencing

We prepared a total of 16 cDNA libraries from *dys-1(eg33)* mixed stage, DP1 mixed stage, DP1 PRE1, DP1 PRE2, DP1 POST1, DP1 POST2, DP2 mixed stages, DP2 PRE1, DP2 PRE2, DP2 POST1, DP2 POST2, *wt* PAP mixed stages, *wt* PAP PRE1, *wt* PAP PRE2, *wt* PAP POST1 and *wt* PAP POST2. Each cDNA library was prepared using 100 ng of precipitated RNAs. cDNA library preparation was performed using the SPIA (Single Primer Isothermal Amplification) technology (IntegenX and NuGEN, San Carlos, CA) as previously described (31, 32). cDNA was then sheared using a Covaris S220 system (Covaris, Woburn, MA), and sample-specific barcodes were sequenced using the HiSeq platform (Illumina, San Diego, CA). We obtained ~60–90 M mappable reads (1 × 75) each dataset.

### Bioinformatics analysis

#### Raw reads mapping

The raw reads were demultiplexed using their unique tissue-specific barcodes and converted to FASTQ files. Unique datasets were then mapped to the *C. elegans* gene model WS250 using the Bowtie 2 algorithm (63) with the following parameters—local -D 20 -R 3 -L 11 -N 1 -p 40—gbar 1 -mp 3. Mapped reads were further converted into a bam format and sorted using SAMtools software using generic parameters (64).

#### Cufflinks/cuffdiff analysis

Expression levels of individual transcripts were estimated from the bam files by using Cufflinks software (65). We calculated the fragment per kilobase per million bases (FPKM) number obtained in each experiment and performed a pairwise with other tissues using the Cuffdiff algorithm (65). We then used the median FPKM value ≥1 between each replicate as a threshold to define positive gene expression levels. The results are shown in Additional File 1: Supplementary Material, Tables S1–S3. Additional File 1: Supplementary Material, Table S4 was compiled using scores produced by the Cuffdiff algorithm (65) and plot using the CummeRbund package.

### RT-PCR experiment for detection of *dys-1* transcripts in *dys-1* strains

N2, *dys-1(eg33)* and *dys-1(cx18)* strains were grown on NGM plates as mixed populations until 200 μl pellets were obtained for each strain. These pellets were subjected to total RNA extraction and DNAse I treatment using the Direct-zol RNA Mini-Prep Plus kit (Zymo Irvine, CA #R2051) according the protocol for tissue samples detailed in the product literature. 200 ng of the RNA obtained was used in cDNA synthesis. About 1 μl of synthesized DNA was used from each cDNA sample to perform PCRs to amplify regions of the *dys-1* gene from N2, *dys-1(eg33)* and *dys-1(cx18)* samples. The following primer sequences were used to detect the presence or absence of *dys-1* transcripts and as a PCR control, respectively: *dys-*1_F: CGGCAAGAAGACAATTGCTCAAA, *dys-*1_R: TCCTCATGAGCATTCAGCTCCG, *myo-2*_F: GGAGTGCTACCGATTGGTTGCCG, *myo-2*_R: CTTGTTCACCCATTCGTTTCCGACC. PCR products were subjected to Sanger sequencing using the following primer: *dys-*1_F: CGGCAAGAAGACAATTGCTCAAA. The primers used for the RT-PCR overlap the exon containing the cx18 mutation.

### Kaplan–Meier survival curve assays

Survival curves were performed as previously described (52, 66). Briefly, we prepared NGM plates each containing 330 μl of 150 mm FUDR (Sigma Life Sciences, Darmstadt, Germany). These plates were seeded with OP50–1 that was UV inactivated prior to plating worms to minimize contamination. All worm strains used in survival curves were synchronized with bleach. We plated 25 L4 worms from each strain per plate, each across three replicates, and stored in 18°C incubators for the duration of the experiment. For each time point, the plates were recovered, and worms were visually inspected and counted directly in the plate using a dissection stereomicroscope (Leica S6E) and a cell counter. The strains were scored for survival every 48 h, with survival being defined as pharyngeal pumping or the ability to move the head in response to prodding by a worm pick. Each plate was anonymized and scored twice.

### RNAi feeding screens at semi-permissive temperatures

RNAi plates were made by adding IPTG to NGM plates to a final concentration of 1 mm. Plates were allowed to dry at room temperature for 5 days before seeding with bacteria. The desired HT115 bacterial cultures were inoculated from glycerol stocks sourced from the Ahringer library (67) and grown in 3 ml LB cultures containing ampicillin (10 μg/ml) and tetracyline (12.5 μg/ml) and grown at 37°C for 16 h (68). Dry NGM plates containing IPTG were then seeded with 75 μl of bacterial culture and left at room temperature to induce dsRNA production overnight for 16 h. RNAi feeding experiments were performed using the temperature-sensitive strains PD4605 (*hlh-1(cc561)*) and LS587 (*hlh-1(cc561); dys-1(cx18)*). Strains were synchronized with bleach as previously described (69) and eggs were incubated at permissive temperature (15°C) until populations reached the L4 stage. Young adults were then plated on NGM plates seeded with the desired RNAi clone and containing 1 mm IPTG, with five worms per plate, and incubated at semi-permissive temperature (18°C) for 24 h to allow young adults to lay eggs. Plates were then recovered, adult worms were removed, and plates containing eggs were returned to semi-permissive temperature to incubate for 24 h on RNAi plates. Plates were then scored for gross defects in body morphology and resulting paralysis. We scored approximately 100 worms per plate across five replicate plates for each strain and each gene tested (67). Each plate was scored twice. The *dys-1* RNAi clone overlaps the Exon 20 of the F15D3.1a gene.

### Preparation of the *dyb-1* clone

The following primers were used to add SacII and SpeI cut sites to the ORF of *dyb-1*:

*dyb-1* F: TCTTCTACTAGTATGTTGTGGTCAAATGGTGG.

*dyb-1* R: CATCATCCGCGGGAAGCCATTGATTGTTACGCC.

The generated PCR fragment was ligated into pDONR221 (Invitrogen) containing the sequence for GFP at the 3′ end. The *dyb-1::GFP* transgene was then moved into the second position of the destination vector pCFJ150 (36), which also contained the *myo-3* promoter and *unc-54* 3′ UTR in first and third positions, respectively.

### RNAi feeding screens on SJ4103 and SJ4103;*cx18* strains

RNAi plates were made by adding IPTG to NGM plates to a final concentration of 1 mm. Plates were allowed to dry at room temperature for 5 days before seeding with bacteria. The desired HT115 bacterial cultures were inoculated from glycerol stocks sourced from the Ahringer library (67) and grown in 3 ml LB cultures containing ampicillin (10 μg/ml) and tetracyline (12.5 μg/ml) and grown at 37°C for 16 h (68). Dry NGM plates containing IPTG were then seeded with 75 μl of bacterial culture and left at room temperature to induce dsRNA overnight for 16 h. SJ4103 and *SJ4103;cx18* strains were synchronized with bleach as previously described (69) and eggs were incubated at room temperature until both populations reached the L4 stage. L4 worms were then moved to IPTG plates seeded with the appropriate RNAi clone. Plates were stored at room temperature until F1 worms reached the young adult stage and were then isolated for fluorescent imaging.

### Fluorescent imaging and analysis of SJ4103 strains

The SJ4103 and SJ4103;*cx18* fluorescent strains were cultured on standard NGM plates at room temperature. To obtain worms at distinct developmental stages, SJ4103 and SJ4103;*cx18* strains were synchronized with bleach. Worms subjected to RNAi feeding were selected for imaging as young adults. Worms were placed on 2% agarose pads and immobilized in 1 mm levamisole. Fluorescent images were taken using a Leica DM13000B microscope and analyzed using ImageJ software (developed by Wayne Rasband at the National Institutes of Health, available at http://rsbweb.nih.gov/ij/).

## Supplementary Material

Supplementary_Figures_ddaa055

Supplementary_table_S3_ddaa055

Supplementary_table_S4_ddaa055

Supplementary_table_S5_ddaa055

## Data and Material Availability

Raw reads were submitted to the NCBI Sequence Read Archive (http://trace.ncbi.nlm.nih.gov/Traces/sra/) under the SRP Study Accession # PRJNA614589. The results of our analyses are available in Excel format as Supplementary Material, Table S3.
