# Supplementary material for: Transcriptome changes during the initiation and progression of Duchenne muscular dystrophy in Caenorhabditis elegans
Source: Hum Mol Genet. 2020 Mar 30;29(10):1607–23. doi: 10.1093/hmg/ddaa055 (PMC7322572; doi:10.1093/hmg/ddaa055)
Supplement: Supplementary_Figures_ddaa055 [file supplementary_figures_ddaa055.pdf]

Additional File 1 for:

Transcriptome changes during the initiation and progression of Duchenne Muscular Dystrophy in *C. elegans*

Heather Hrach[1,2], Shannon O'Brien[2], Hannah Steber[3], Jason Newbern[4], Alan Rawls[4] and Marco Mangone[2*]

* To whom correspondence should be addressed. Tel: (480) 965-7957; Email: mangone@asu.edu

This PDF includes:

Figs. S1-S7

Table S1 - Summary of results from PAT-Seq after deep sequencing.

Table S2 - Summary of genes detected in this study.

Table of Contents

Additional File 1

Fig. S1: Kaplan-Meier Survival Analysis and validation of filtration experiments………………………………………………………………………....3

Fig. S2 : Sequencing results…………………………………………………………….4

Fig. S3: Validation and comparison with other studies…...............................5

Fig. S4: Differential gene expression analysis in PRE- and POST-symptomatic strains…………………………………………………………….6

Fig. S5: *dyb-1* assembles within the DGC in the absence of *dys-1* …………..7

Fig. S6: Splicing defects in *dys-1*(*eg33*)…………………………………………………..8

Fig. S7: Spearman correlation of read counts in DP1 (cx18) and DP2 (eg33) PRE and POST datasets……………………………………..9

Table S1: Summary of results after deep sequencing…………………………....10

Table S2: Summary of genes detected in this study…………………………..…..11

Fig. S1

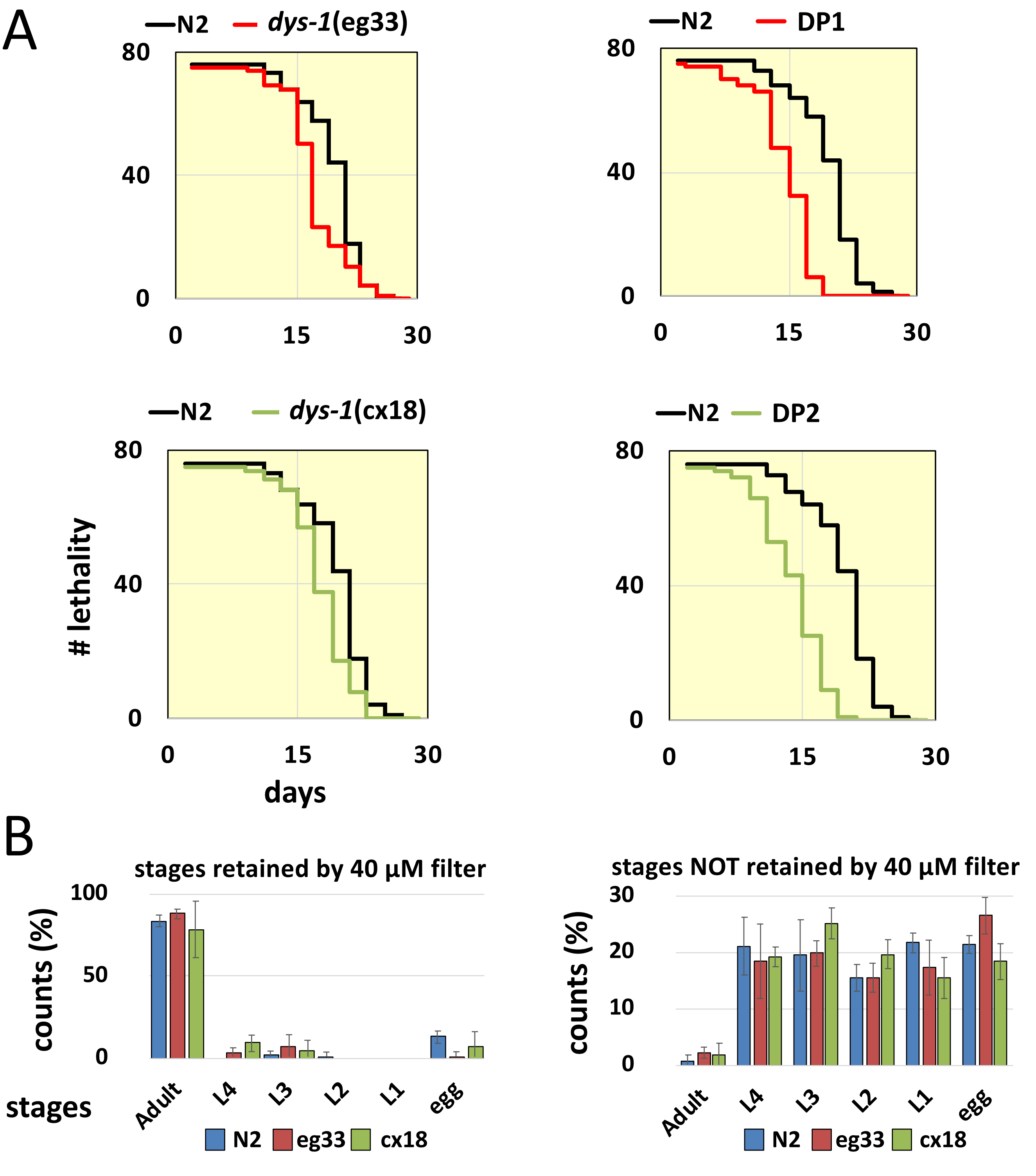

Fig. S1. Kaplan-Meier Survival Analysis and validation of filtration experiments. (A) Survival curves were performed on *dys-1(cx18)*, *dys-1(eg33*), DP1, and DP2 to confirm the effects of dystrophin deficiencies and the PAP cassette on lifespan. Each graph represents the average percent lethality for 75 worms per control and experimental strain. Left Panels compare both i*dys-1* strains to *wt* strains. Right Panels compare both *dys-1* strains after crossing with *wt* strains containing our PolyA-pull (PAP) constructs. (B) A similar number of N2, *dys-1(eg33)*, *dys-1(cx18)* worm strains in each developmental stage were able to pass through our 40 µM filter. The left graph indicates the quantity of each strain and their respective developmental stage retained by the filter (n=190). Right panel indicates the quantity of each strain and developmental stage that passed through the filter (n=676).

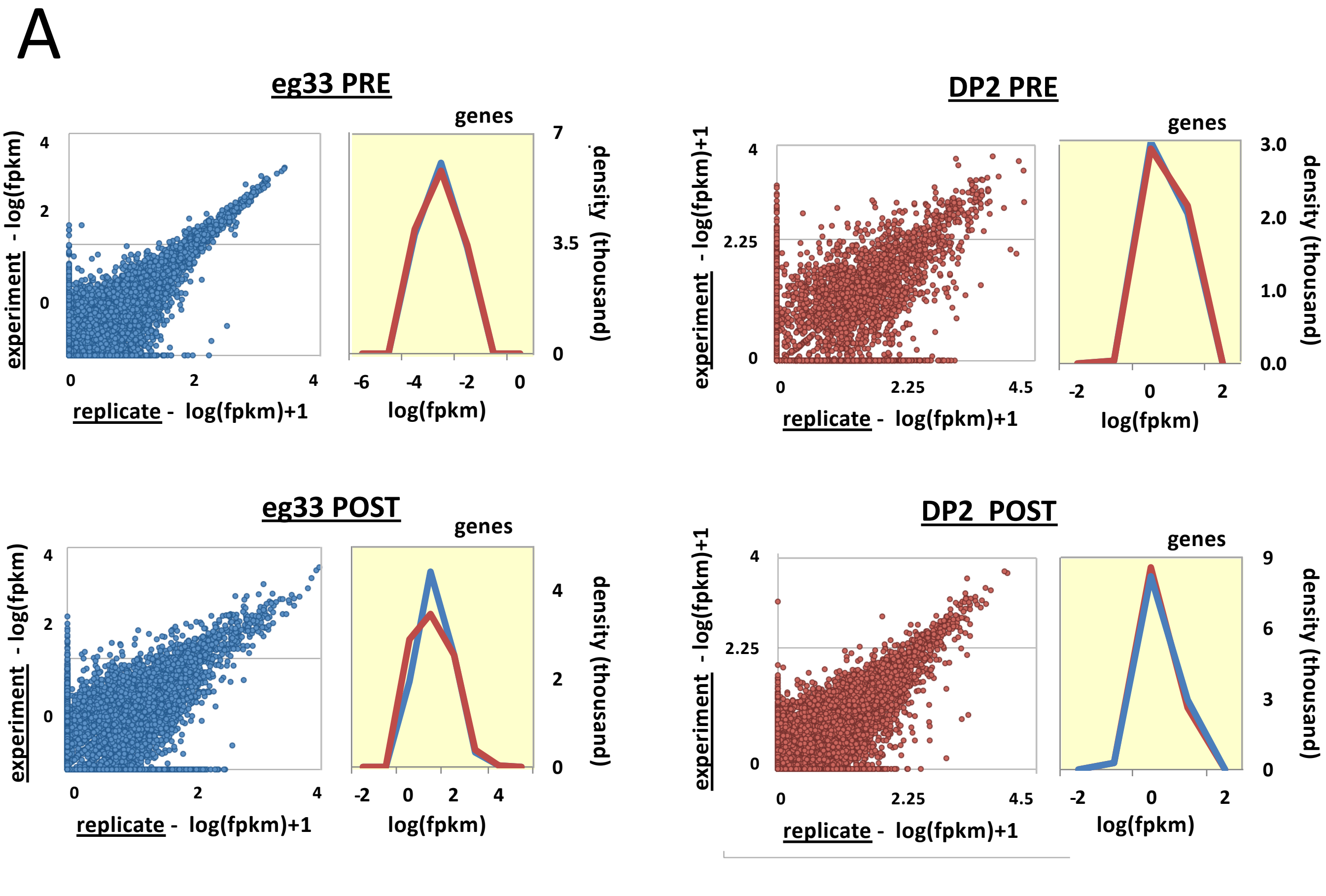

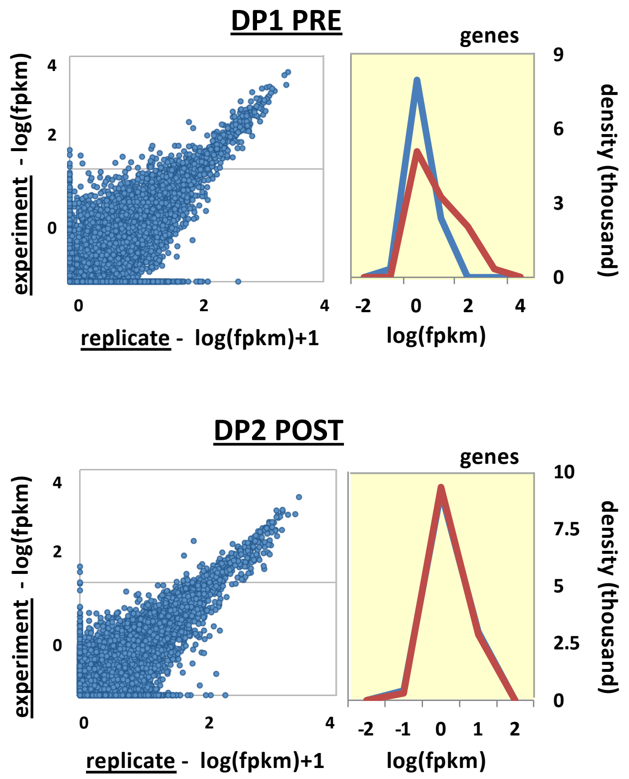

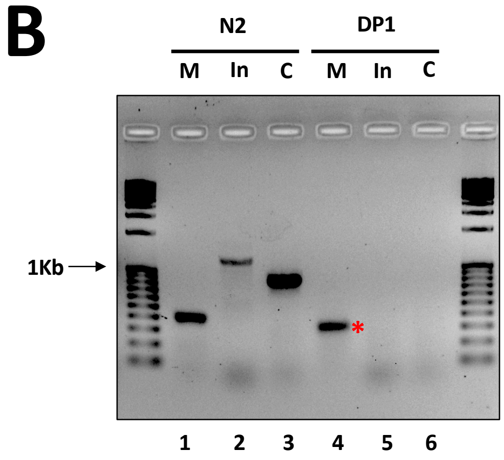

Fig. S2: Sequencing results. (A) Left panels: Scatter plot of mapped genes from eg33 PRE and POST, DP1 PRE and POST and DP2 PRE and POST datasets displayed by fpkm value detected in each replicate on a logarithmic (log10) scale, to highlight the similarity of detection between replicates. Right panels: The distribution of the fpkm values in experiment (red) and replicate (blue) samples for each dataset. The plots were generated using the cummeRbund package v. 2.0. (B) Quantification of the specificity and sensitivity of the pull-down experiments using genomic PCR (lanes 1, 2, and 3) and RT-PCR (lane 4, 5 and 6). Using immunoprecipitation we successfully isolate the muscle specific gene *myo-3* (lane 4) (*) from RNA from our DP1 strain, but not the intestine specific gene *ges-1* (lane 5) and the cuticle specific gene dpy-7 (lane 6). Lane 1, 2 and 3 show the expected band sizes of the genes tested.

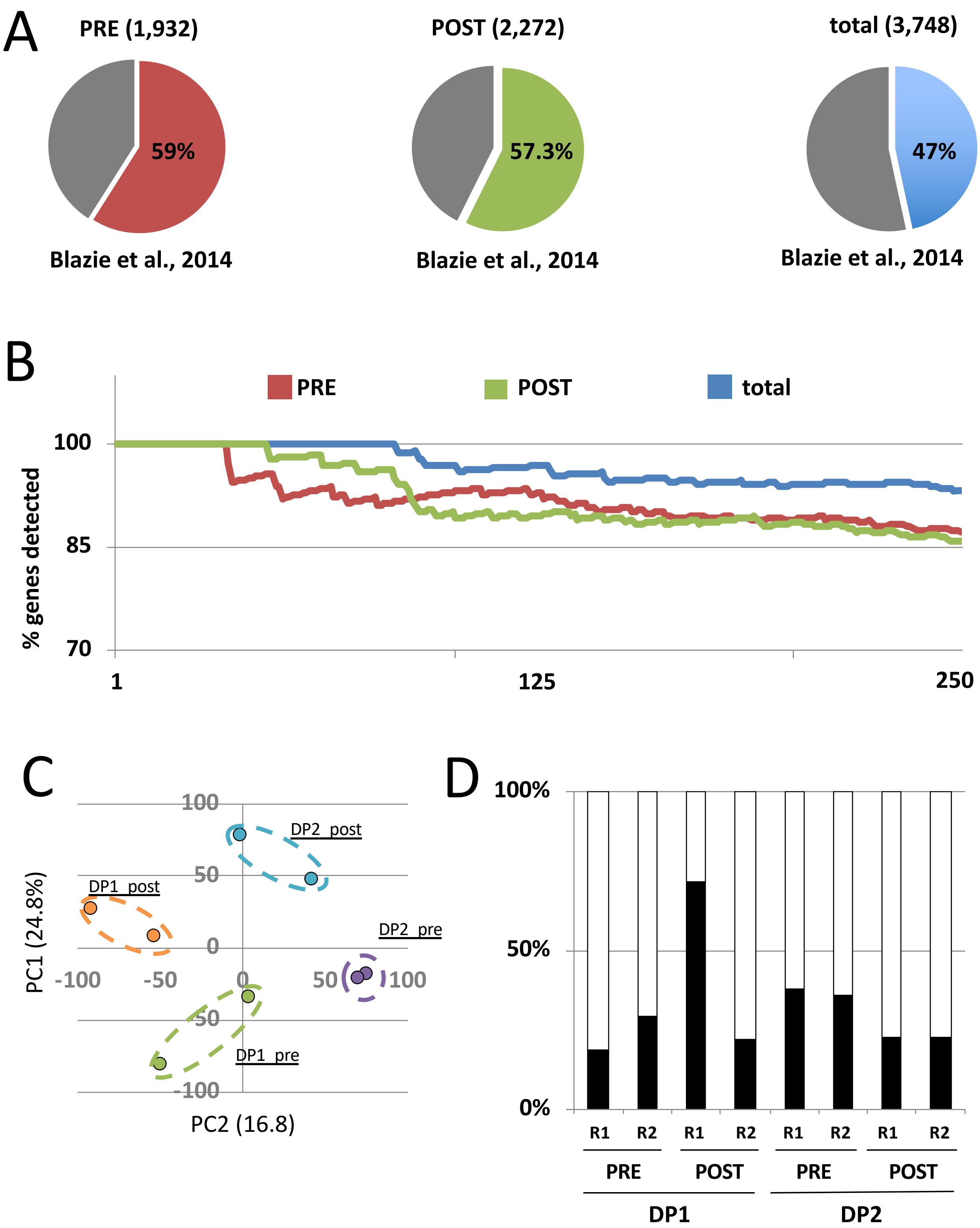

Fig. S3: Validation and comparison with other studies. (A) Comparative analysis of genes detected in our study versus body muscle-specific datasets from Blazie et al., 2014. The number of genes overlapping our PRE, POST, and a combined dataset (total) are summarized. (B) Genes identified in this study and the muscle-specific dataset from Blazie et al., are ranked by fpkm value, and the top 250 genes from both datasets are compared. (C) Principal Component Analysis (PCA) shows a high correlation among each duplicate within our datasets. (D) For this study, we have used the top ~30-40% positive hits produced by Cufflinks. Black bars: % of reads used for each dataset in this study. White bars: % of reads discarded. R1 and R2: Replicate 1 and Replicate 2.

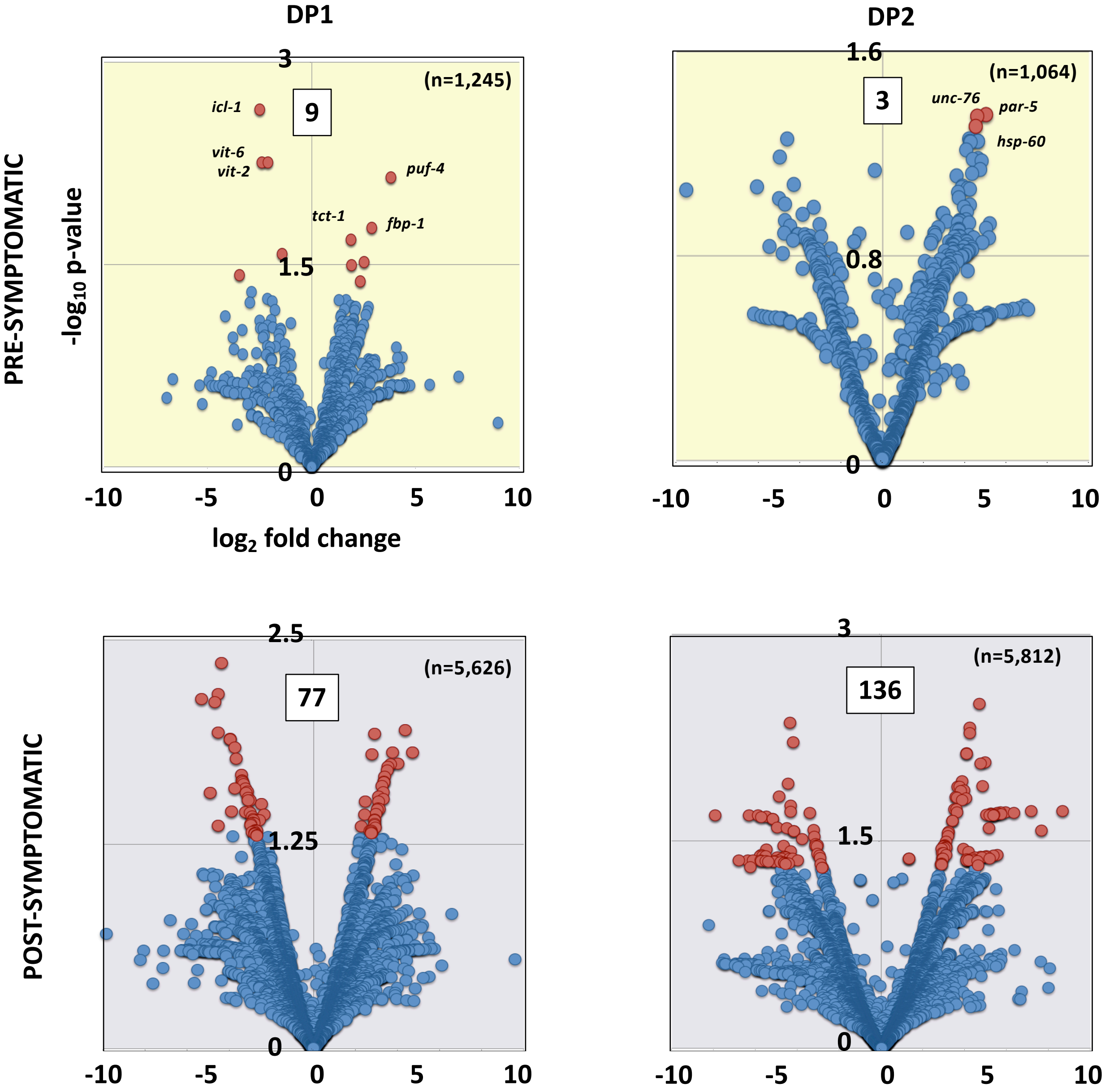

Fig. S4: Differential gene expression analysis in PRE- and POST- symptomatic strains. We have studied the changes in gene expression for genes detected in both *wt* PAP, DP1, and DP2 strains. The volcano plots show the changes of gene expression between each tissue (p-value versus fold-change). The total number of genes that significantly switch between PRE and POST symptomatic ($p<0.05$) are shown in red and boxed. The top genes identified in our PRE dataset are named in the chart. The density for the genes detected in the POST dataset prevents individual labeling. This analysis has been performed using the SAMtools suite (Li et al., 2009). The complete list of genes produced by this software is shown in Supplemental Table S4.

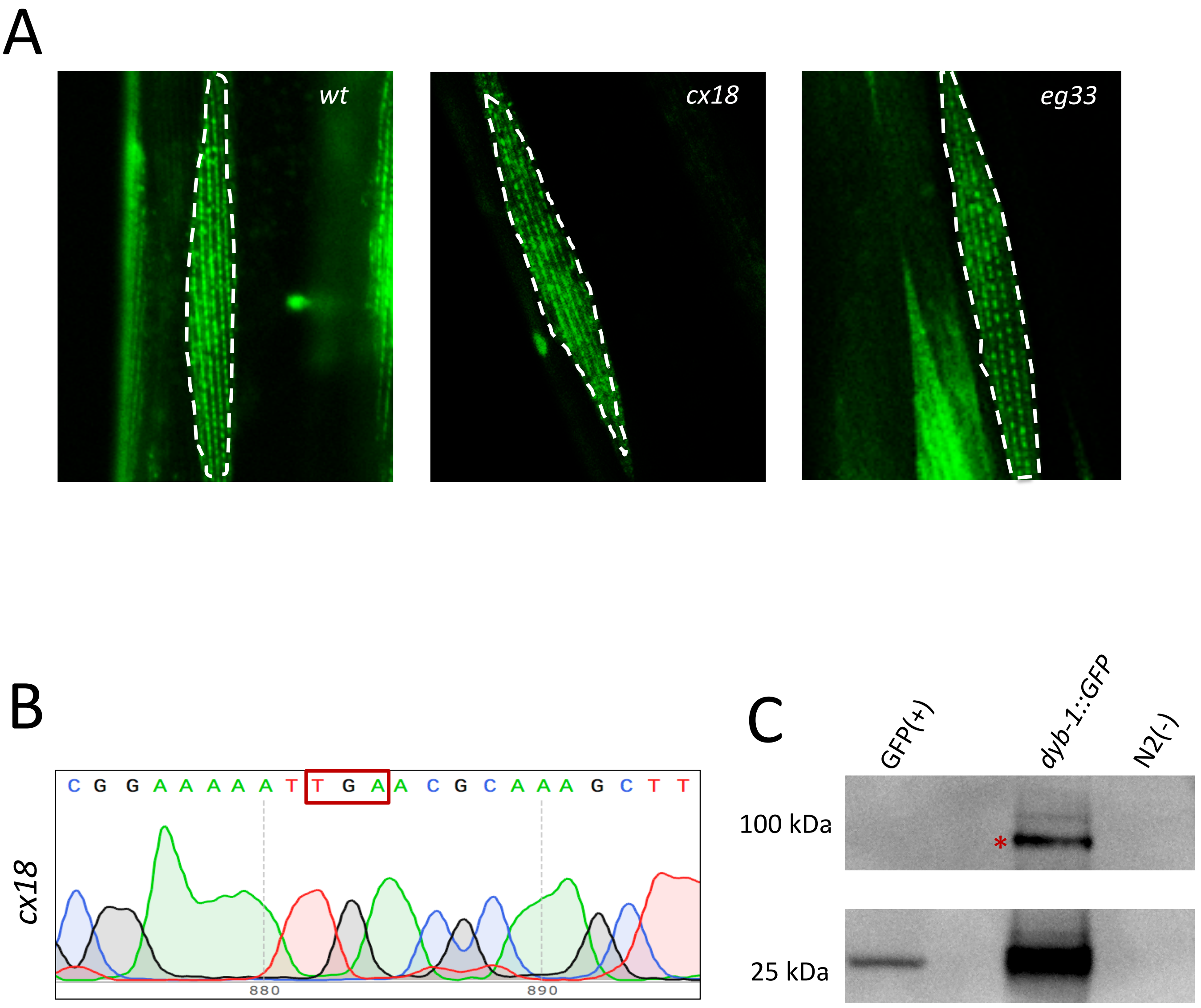

Fig. S5: *dyb-1* assembles within the DGC in the absence of *dys-1*. (A)Fluorescent images showing localization pattern of *dyb-1::GFP* along muscle fibres in *wt* and *dys-1*(cx18) genetic backgrounds. A single muscle cell in each strain is outlined in white to indicate regions being compared between images. (B) Trace file depicts the presence of the expected premature stop codon in the *cx18; dyb-1::GFP* strain. (C) Western blot results in right panel show predicted protein size of *dyb-1::GFP* fusion at 96 kDa (highlighted with a red asterisk).

A

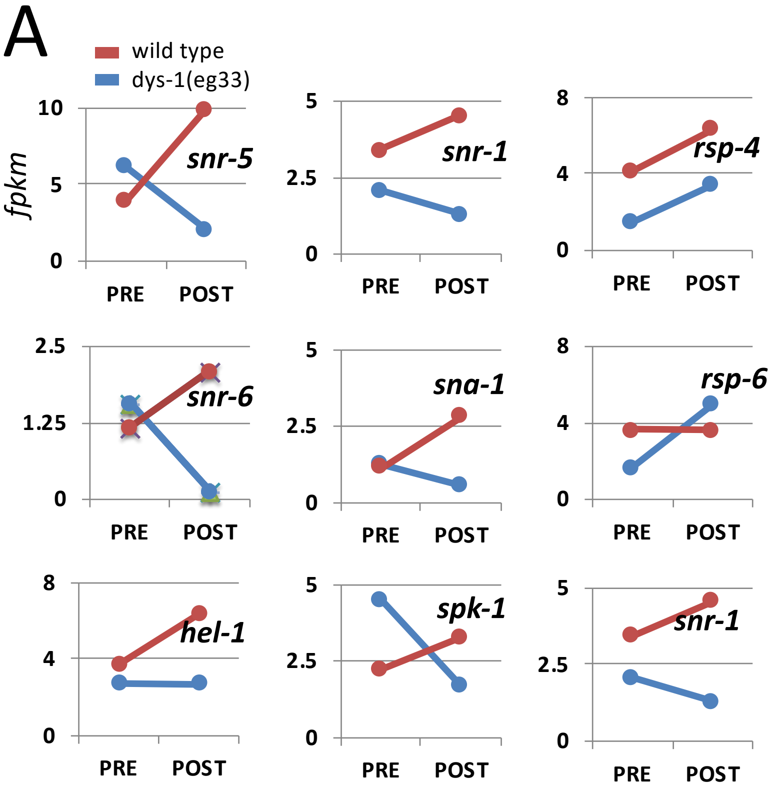

B

n=26,692

# of novel splice junctions				
	known	novel		
		inclusion	exclusion	aberrant
PRE	14,131	192	73	474
POST	11,040	241	127	939
total	25,171	433	200	1,413

C

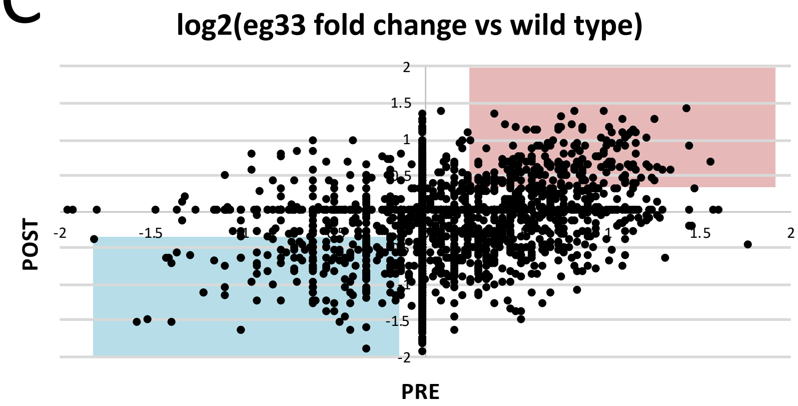

Fig. S6: Splicing defects in *dys-1(eg33)*. A) Change in gene expression identified in 9 snRNPs and SR genes from our PRE and POST datasets. B) total number of novel splice junction identified in this study. C) Analysis of splice junction usage in the DP1 dataset. The graph illustrates the changes in splice junction abundance. The x-axis represents the fold-change of the number of reads for each splice junction comparing the PRE dataset to wild type, while the y-axis represent the fold change obtained when comparing DP1 POST dataset to wild type. Splice junctions with more than 2-fold enrichment in both datasets are highlighted in red, while splice junctions with 2-fold depletion in both datasets are highlighted in blue.

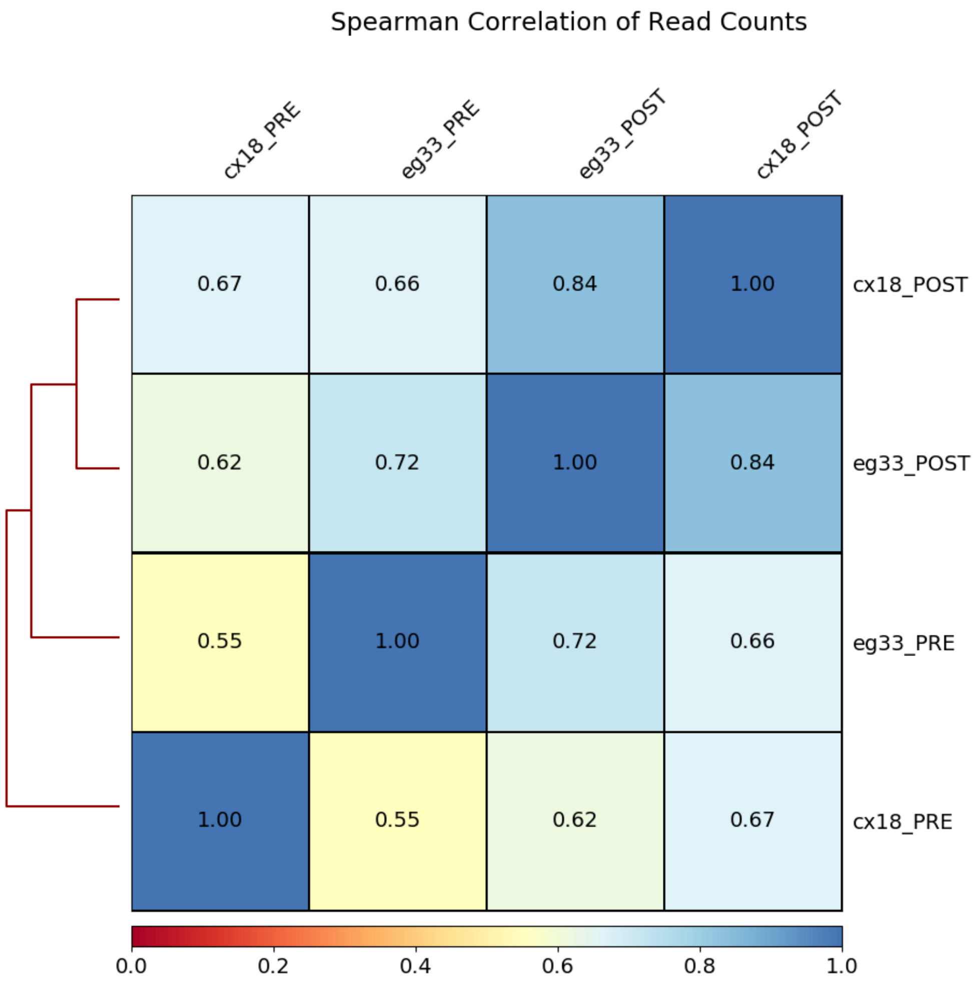

Fig. S7: Spearman correlation of read counts in DP1 (cx18; Pmyo-3 PAP) and DP2 (eg33; Pmyo-3-PAP) PRE and POST datasets. The chart shows an overall similarity between the DP1 and DP2 datasets. This analysis was performed using the DeepTools 2.1.0 suite (Ramirez et al., 2014).

Sample			Total reads	Mapped (%)	Not mapped
dys-1(eg33)	PRE	experiment	61,331,245	16,458,692 (27)	44,872,553
		replicate	46,677,723	17,812,390 (38.1)	28,865,333
	POST	experiment	60,617,365	55,397,232 (91.39)	5,220,133
		replicate	45,052,554	40,638,981 (90.2)	4,413,573
PAP	PRE	experiment	60,136,874	25,824,737 (43)	34,312,137
		replicate	38,934,890	15,628,961 (40)	23,305,929
	POST	experiment	47,138,436	13,403,525 (28.5)	33,734,911
		replicate	35,399,372	15,437,939 (44)	19,961,433
DP1	PRE	experiment	74,708,361	6,519,607 (9)	68,188,754
		replicate	80,851,313	8,513,602 (10.5)	72,337,711
	POST	experiment	85,543,247	60,585,159 (70.8)	24,958,088
		replicate	76,252,221	49,950,579 (65.5)	26,301,642
DP2	PRE	experiment	86,493,693	16,895,535 (19.5)	69,598,158
		replicate	27,624,484	2,174,470 (8)	25,450,014
	POST	experiment	36,061,335	23,851,357 (66.1)	12,209,978
		replicate	29,015,306	17,706,345 (61)	11,308,961

Table S1: Summary of results from PAT-Seq after deep sequencing.

Sample			Genes	
dys-1(eg33)	PRE	experiment	3,394	2,603*
		replicate	3,416	
	POST	experiment	2,951	1,738*
		replicate	2,957	
PAP	PRE	experiment	2,864	1,712*
		replicate	2,996	
	POST	experiment	3,487	2,187*
		replicate	3,353	
DP1	PRE	experiment	2,078	1,100*
		replicate	2,176	
	POST	experiment	3,020	1,950*
		replicate	2,636	
DP2	PRE	experiment	2,415	1,640*
		replicate	2,454	
	POST	experiment	3,005	2,266*
		replicate	2,885	

Table S2: Summary of genes detected in this study. Raw reads derived from the mRNA libraries on the Illumina Hi-Seq Instrument, mapped to the C. elegans WS250 genome annotation. Genes marked with an asterisk correspond to genes detected in both biological replicates (fpkm>=4)
